# Genomewide Profiling of the Enterococcus faecalis Transcriptional Response to Teixobactin Reveals CroRS as an Essential Regulator of Antimicrobial Tolerance

**DOI:** 10.1128/mSphere.00228-19

**Published:** 2019-05-08

**Authors:** Rachel L. Darnell, Melanie K. Knottenbelt, Francesca O. Todd Rose, Ian R. Monk, Timothy P. Stinear, Gregory M. Cook

**Affiliations:** a Department of Microbiology and Immunology, University of Otago, Dunedin, New Zealand; b Maurice Wilkins Centre for Molecular Biodiscovery, University of Auckland, Auckland, New Zealand; c Department of Microbiology and Immunology, Peter Doherty Institute for Infection and Immunity, University of Melbourne, Melbourne, Australia; University of Nebraska Medical Center

**Keywords:** CroRS, *Enterococcus*, RNA sequencing, teixobactin, antimicrobial resistance, antimicrobial tolerance, mechanisms of resistance

## Abstract

Teixobactin is a new antimicrobial of significant interest. It is active against a number of multidrug-resistant pathogens, including Staphylococcus aureus and Enterococcus faecalis, with no reported mechanisms of teixobactin resistance. However, historically, mechanisms of resistance always exist and arise upon introduction of a new antimicrobial into a clinical setting. Therefore, for teixobactin to remain effective long term, we need to understand how mechanisms of resistance could develop. Here we demonstrate that E. faecalis shows a remarkable intrinsic tolerance to high concentrations of teixobactin. This is of critical importance, as antimicrobial tolerance has been shown to precede the development of antimicrobial resistance. To identify potential pathways responsible for this tolerance, we determined the genomewide expression profile of E. faecalis strain JH2-2 in response to teixobactin using RNA sequencing. A total of 573 genes were differentially expressed (2.0-fold log_2_ change in expression) in response to teixobactin, with genes involved in cell wall biogenesis and division and transport/binding being among those that were the most upregulated. Comparative analyses of E. faecalis cell wall-targeting antimicrobial transcriptomes identified CroRS, LiaRS, and YclRK to be important two-component regulators of antimicrobial-mediated stress. Further investigation of CroRS demonstrated that deletion of *croRS* abolished tolerance to teixobactin and to other cell wall-targeting antimicrobials. This highlights the crucial role of CroRS in controlling the molecular response to teixobactin.

**IMPORTANCE** Teixobactin is a new antimicrobial with no known mechanisms of resistance. Understanding how resistance could develop will be crucial to the success and longevity of teixobactin as a new potent antimicrobial. Antimicrobial tolerance has been shown to facilitate the development of resistance, and we show that E. faecalis is intrinsically tolerant to teixobactin at high concentrations. We subsequently chose E. faecalis as a model to elucidate the molecular mechanism underpinning teixobactin tolerance and how this may contribute to the development of teixobactin resistance.

## Abstract (precis)

Teixobactin is a new antimicrobial with no known mechanisms of resistance. Understanding how resistance could develop will be crucial to the success and longevity of teixobactin as a new potent antimicrobial. Antimicrobial tolerance has been shown to facilitate the development of resistance, and we show that E. faecalis is intrinsically tolerant to teixobactin at high concentrations. We subsequently chose E. faecalis as a model to elucidate the molecular mechanism underpinning teixobactin tolerance and how this may contribute to the development of teixobactin resistance.

## INTRODUCTION

The emergence of multidrug-resistant pathogens has rendered standard antimicrobial treatments ineffective, allowing infections to persist and proliferate. This is compounded by our lack of understanding of how antimicrobial resistance develops and a severe deficiency of new antimicrobials to treat resistant infections. Teixobactin is the first new class of antimicrobial to be discovered in decades and has proven efficacy against multidrug-resistant pathogens, such as vancomycin-resistant enterococci (VRE), methicillin-resistant Staphylococcus aureus (MRSA), and Mycobacterium tuberculosis (1). Teixobactin is a unique depsipeptide antimicrobial consisting of 11 amino acids, including methylphenylalanine, enduracididine, and four d-amino acids (1). It was isolated from a new species of Gram-negative betaproteobacteria, Eleftheria terrae, using the multichannel iChip device (1). The potency of teixobactin stems from its ability to dually target the essential pyrophosphate-saccharide (PP-sugar) moiety of the cell wall precursors lipid II and lipid III, inhibiting both peptidoglycan and cell wall teichoic acid biosynthesis (1). The bactericidal mode of action of teixobactin has been identified in S. aureus, with inhibition of cell wall teichoic acids leading to a dysregulation of cell wall autolysins, resulting in cell lysis and death (1, 2). However, this remains to be determined in other bacterial pathogens.

There are no reported mechanisms of teixobactin resistance, with the producer strain (and other Gram-negative bacteria) being innately resistant, likely due to the inability of teixobactin to penetrate the outer membrane (1). This is in contrast to other antimicrobial producer species, such as Bacillus licheniformis, a Gram-positive bacterium which requires a resistance cassette to provide protection against bacitracin production (3, 4). The absence of a known naturally occurring resistance cassette therefore makes teixobactin a promising new antimicrobial for the treatment of infections caused by multidrug-resistant organisms. However, historically, resistance mechanisms always exist and appear upon introduction of an antimicrobial into a clinical setting (5, 6). Therefore, for teixobactin to remain effective long term, we need to understand how mechanisms of resistance could develop.

Bacterial stress response systems can act as determinants of antimicrobial resistance (7). When bacteria are challenged with cell wall-acting antimicrobials, they encounter a number of cellular stresses, including oxidative stress, nutrient limitation, and cell envelope stress (8–11). These stresses elicit a variety of specific and highly regulated adaptive responses that not only protect the bacteria from the offending stress but also promote changes in the cell that can impact innate susceptibility to additional antimicrobials (7). Antimicrobial network biology involves bioinformatic approaches which use high-throughput genetic screening or gene expression profiling, i.e., RNA sequencing (RNA-seq), to explore the different response layers of bacteria to different antimicrobial treatments (12). These high-throughput methods allow monitoring of the global changes in gene expression and can provide important insights into how groups of genes interact in response to antimicrobial stress (12–14).

Enterococci are opportunistic pathogens localized to the gastrointestinal tract of humans and animals (15). They are one of the leading causes of hospital-acquired infection, and infections caused by these organisms are notoriously difficult to treat (16). Antimicrobial tolerance was recently described to be an essential precursor to the development of ampicillin resistance in Escherichia coli, and it has long been known that enterococci are intrinsically tolerant to cell wall-targeting antimicrobials (17, 18). Previous studies have identified SodA, a superoxide dismutase, to be a key component of antimicrobial tolerance in Enterococcus faecalis (19, 20). However, SodA is not differentially expressed in response to cell wall-targeting antimicrobials; therefore, we hypothesize that there may be more than one mechanism of antimicrobial tolerance in E. faecalis (10).

The aim of this study was to identify and characterize potential pathways of teixobactin tolerance in E. faecalis using RNA-seq analysis. We report on the teixobactin-induced transcriptome of E. faecalis and isolation of the cell wall stress response two-component system (TCS) CroRS as an essential regulator of teixobactin tolerance and a potential contributor to the development of teixobactin resistance.

## RESULTS AND DISCUSSION

### E. faecalis displays tolerance to teixobactin at high concentrations.

**FIG 1:**
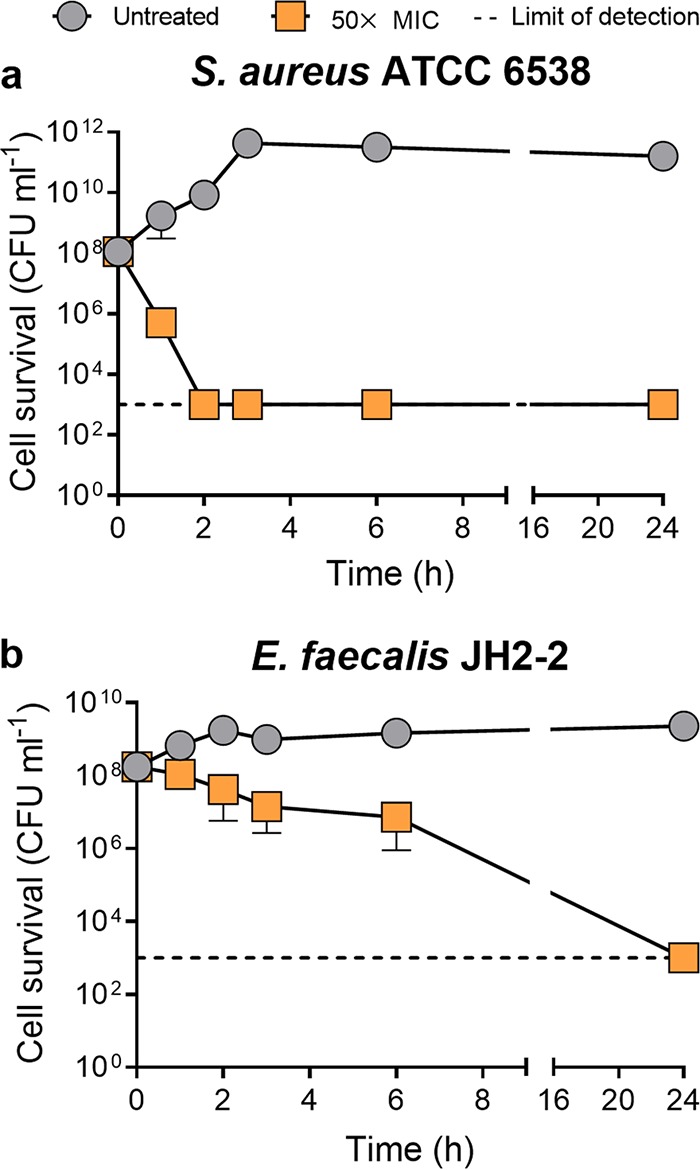
Time-dependent kill kinetic assay of S. aureus and E. faecalis upon teixobactin challenge. Strains were grown to mid-exponential phase (5 × 10^8^ CFU ml^−1^) and untreated or challenged with 50× MIC of teixobactin. Cell survival (number of CFU ml^−1^) was measured at time zero and 1, 2, 3, 4, 6, and 24 h postchallenge. Results are the mean ± SD (data are for biological triplicates).

Antimicrobial tolerance is the ability of an organism to survive for extended periods of time in the presence of high antimicrobial concentrations, even when growth is inhibited. Importantly, antimicrobial tolerance is a critical preliminary factor in the acquisition of antimicrobial resistance, and it has long been known that enterococci are intrinsically tolerant to cell wall-targeting antimicrobials (17, 18). A comparison of E. faecalis JH2-2 and S. aureus ATCC 6538 MICs and minimum bactericidal concentrations (MBCs) showed that while the growth of both species was inhibited at 2 μg ml^−1^ (MIC), E. faecalis showed remarkable tolerance to cell killing by teixobactin with an MBC of 16 μg ml^−1^, compared to an MBC of 2 μg ml^−1^ for S. aureus (Table 1). This tolerance is also consistent with that to other cell wall-targeting antimicrobials (Table 1). This was confirmed by time-dependent kill kinetic assays, which demonstrated that a culture of S. aureus (5 × 10^8^ CFU ml^−1^) was effectively sterilized by teixobactin at 50× MIC with a 5-log reduction in the number of CFU ml^−1^ to the lowest detectable level (1 × 10^3^ CFU ml^−1^) in 2 h, while E. faecalis remained tolerant at >24 h postchallenge at the same concentration (Fig. 1). Ling et al. were unable to generate resistance to teixobactin using spontaneous mutagenesis in S. aureus (1). As tolerance has been shown to precede the development of resistance, we hypothesize that a lack of tolerance to teixobactin in S. aureus may hinder the bacterium’s ability to acquire mutations that could potentially confer resistance (17).

**TABLE 1:** Teixobactin MICs and MBCs for S. aureus and E. faecalis^*a*^

Strain^*b*^	Antimicrobial	MIC (μg ml^−^^1^)	MBC (μg ml^−^^1^)
S. aureus	Teixobactin	2	2
E. faecalis	Teixobactin	2	16
E. faecalis	Vancomycin	1	>128
E. faecalis	Bacitracin	32	64
E. faecalis	Ampicillin	0.5	2
E. faecalis	Penicillin G	2	2
E. faecalis	Daptomycin	2	4

a Mean MICs and MBCs for at least three biological replicates are reported.

b S. aureus strain ATCC 6538 and E. faecalis strain JH2-2.

SodA is a superoxide dismutase responsible for the conversion of superoxide (O_2_^−^) to hydrogen peroxide (H_2_O_2_) and has previously been associated with antimicrobial tolerance in enterococci (19). However, *sodA* gene expression does not appear to be significantly up- or downregulated in response to other cell wall-targeting antimicrobials; we therefore hypothesized that there could be additional pathways that regulate teixobactin tolerance in E. faecalis (10).

### Global gene expression profiling of E. faecalis in response to teixobactin.

**FIG 2:**
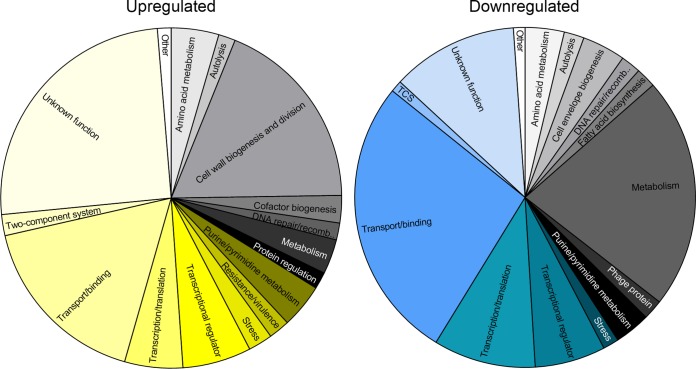
Pie charts showing the distribution of gene ontologies up- and downregulated in response to teixobactin. recomb., recombination.

To identify potential pathways of intrinsic teixobactin tolerance, we performed global gene expression profiling of E. faecalis in response to teixobactin. E. faecalis JH2-2 was grown microaerobically (130 rpm) to mid-exponential phase (optical density at 600 nm [OD_600_], 0.5) and challenged with 0.25× MIC of teixobactin (0.5 μg ml^−1^) for 1 h (see Fig. S1 in the supplemental material). Challenge at this concentration ensured a teixobactin-induced response while minimizing growth inhibition (30% inhibition), thereby reducing changes in growth rate-related gene expression. A total of 573 genes were differentially expressed (2.0-fold log_2_ change in expression) in response to teixobactin challenge, with 306 being upregulated and 268 being downregulated (Tables S1 and S2). These results were confirmed by quantitative real-time PCR (Fig. S2; Table S3 and S6). Genes were categorized into gene ontologies to achieve an overall view of pathways up- and downregulated at a global transcriptomic level (Fig. 2). Excluding genes of unknown function (25.2% upregulated, 12% downregulated), genes involved in cell wall biogenesis and division (18.6%) and transport/binding (17%) were the most frequently upregulated in response to teixobactin (Fig. 2), while genes involved in metabolism (22%) and transport/binding (27%)—specifically, phosphotransfer system (PTS) transporters (11.6%) involved in the uptake of carbon metabolites—were the most frequently downregulated in response to teixobactin (Fig. 2).

10.1128/mSphere.00228-19.1 TABLE S1 Genes upregulated in response to teixobactin in Enterococcus faecalis JH2-2. #, log_2_ fold change (2-fold log_2_ minimum threshold). Download Table S1, DOCX file, 0.07 MB. Copyright © 2019 Darnell et al. 2019 Darnell et al. This content is distributed under the terms of the Creative Commons Attribution 4.0 International license.

10.1128/mSphere.00228-19.2 TABLE S2 Genes downregulated in response to teixobactin in Enterococcus faecalis JH2-2. #, log_2_ fold change (2-fold log_2_ minimum threshold). Download Table S2, DOCX file, 0.06 MB. Copyright © 2019 Darnell et al. 2019 Darnell et al. This content is distributed under the terms of the Creative Commons Attribution 4.0 International license.

10.1128/mSphere.00228-19.3 TABLE S3 A comparison of the differential changes in gene expression for 10 genes in response to teixobactin using qRT-PCR and RNA-seq. #, not detected (ND; below the 2-fold log_2_ threshold in RNA-seq analysis). Download Table S3, DOCX file, 0.01 MB. Copyright © 2019 Darnell et al. 2019 Darnell et al. This content is distributed under the terms of the Creative Commons Attribution 4.0 International license.

10.1128/mSphere.00228-19.7 FIG S1 Optimization of teixobactin concentration for RNA sequencing. E. faecalis JH2-2 was grown to mid-exponential phase (OD_600_, 0.5) and challenged with 0, 0.2, 0.5, and 1 μg ml^−1^ of teixobactin or DMSO at time zero. Growth was measured by determination of the cell density (OD_600_) for 3 h postchallenge. RNA was extracted at 1 h postchallenge. Results are the mean ± SD (data are for technical duplicates). Download FIG S1, TIF file, 0.2 MB. Copyright © 2019 Darnell et al. 2019 Darnell et al. This content is distributed under the terms of the Creative Commons Attribution 4.0 International license.

10.1128/mSphere.00228-19.8 FIG S2 qRT-PCR of E. faecalis JH2-2 gene expression in response to teixobactin. Quantitative real-time-PCR was carried out for 10 genes using E. faecalis JH2-2 cDNA to validate the changes in gene expression observed in the RNA sequencing data. Fold change is represented as a ratio of the mean *C_T_* values normalized to the *C_T_* value for the constitutively expressed EF0013. Results are the mean ± SD (data are for technical triplicates). Download FIG S2, TIF file, 0.1 MB. Copyright © 2019 Darnell et al. 2019 Darnell et al. This content is distributed under the terms of the Creative Commons Attribution 4.0 International license.

The 20 most upregulated genes included genes for three putative autolysins (EF1518 [9.5-fold], EF0443 [8.7-fold], and EF0737 [5.5-fold]), four efflux transporters (EF2986 [6.8-fold], EF2987 [6.3-fold], EF2050 [6.3-fold], and EF1198 [5.7-fold]), a penicillin binding protein (EF0680 [5.4-fold]), and a glutamate-5-kinase (EF0038 [6.2-fold]) involved in cell wall biosynthesis and amino acid metabolism (Table 2). A total of 11 genes with unknown function were also among the most upregulated, including EF1533 (7.9-fold), EF0932 (7.1-fold), and EF1258 (6.0-fold) (Table 2). EF1533 appears to have a role in bacitracin and vancomycin susceptibility, while EF0932 is also highly upregulated in response to the antiseptic chlorhexidine (10, 21). Previous attempts to create an EF1258 deletion mutant suggest that this protein has an essential function (10). Eighteen of the 20 most downregulated genes were involved in carbon metabolism, including 13 transporters (11 PTS transporters), 4 metabolic genes (EF3141 [−9.9-fold], EF3142 [−9.7-fold], EF3140 [−9.4-fold], and EF0413 [−9.7-fold]), and 1 regulator (EF2966 [−9.3-fold]) (Table 2). The remaining two genes, EF2582 (−9.0-fold) and EF2223 (−8.8-fold), encode a chlorohydrolase/aminohydrolase and an ABC transporter, respectively, both of which are of unknown function (Table 2). E. faecalis JH2-2 teixobactin MICs and MBCs were determined in M17 broth supplemented with 0.5% glucose to identify a role for the PTS system in teixobactin susceptibility. However, no significant difference in MICs or MBCs was observed (data not shown).

**TABLE 2:** The 20 most up- and downregulated genes in response to teixobactin in E. faecalis V583 and JH2-2

Gene regulation	Gene in E. faecalis:		Gene name	F/C^*a*^	Function
	V583	JH2-2			
Upregulated	EF1518	1316		9.5	Soluble lytic murein transglycosylase
	EF0443	2523		8.7	Endopeptidase
	EF1533	1329		7.9	Conserved hypothetical protein
	EF0802	545		7.8	DUF3955 domain-containing protein
	EF1665	1454		7.6	Conjugal transfer protein TraX
	EF1231	1016		7.6	Metallophosphoesterase
	EF2896	2418		7.2	DUF3955 domain-containing protein
	EF0932	663		7.1	Hypothetical protein
	EF2986	287		6.8	ABC transporter ATP-binding protein
	EF2050	1817		6.3	Peptide ABC transporter: ATP-binding protein
	EF2987	286		6.3	RND transporter
	EF1532	1328		6.2	Hypothetical protein
	EF0038	2863	*proB*	6.2	Glutamate-5-kinase
	EF2771	2347		6.0	TraX family protein
	EF1258	1042		6.0	Hypothetical protein
	EF2214	1918		5.8	VOC family protein
	EF1198	982		5.7	ABC transporter permease
	EF0737	484		5.5	Amidase
	EF2211	1915		5.4	YxeA family protein
	EF0680	422		5.4	Penicillin binding protein 1A
Downregulated	EF0411	2555		−11.6	PTS mannitol transporter subunit IICB
	EF3139	138		−10.9	PTS sugar transporter subunit IIC
	EF0412	2554		−10.2	PTS mannitol transporter subunit IIA
	EF3141	136		−9.9	2-Hydroxyacid dehydrogenase
	EF2965	2466		−9.9	PTS sugar transporter subunit IIB
	EF3142	135		−9.7	6-Phosphogluconate dehydrogenase
	EF0413	2553		−9.7	Mannitol-1-phosphate 5-dehydrogenase
	EF3213	69		−9.6	PTS mannose transporter subunit IID
	EF3140	137		−9.4	Oxidoreductase
	EF3211	71		−9.4	PTS mannose/fructose/sorbose/*N*-acetylglucosamine subunit IIB
	EF2966	2467		−9.3	MltR-like mannitol-operon transcriptional regulator
	EF2582	2168		−9.0	Chlorohydrolase/aminohydrolase
	EF3138	139		−9.0	PTS mannose transporter subunit IID
	EF2964	2465	*ulaA*	−8.9	PTS ascorbate transporter subunit IIC
	EF2223	1927		−8.8	ABC transporter family
	EF3212	70		−8.7	PTS mannose/fructose/sorbose/*N*-acetylglucosamine subunit IIC
	EF3327	2909		−8.7	Citrate transporter
	EF3210	72		−8.6	PTS mannose/fructose/sorbose/*N*-acetylglucosamine subunit IIA
	EF1031	742		−8.4	PTS sugar transporter subunit IIC
	EF1207	991	*maeP*	−8.4	l-Malate permease

a F/C, log_2_ fold change.

### Cell wall precursors as a target of teixobactin.

Lipid II and lipid III are precursors of peptidoglycan and teichoic acid biosynthesis, and both share the PP-sugar target moiety of teixobactin. Inhibition of these biosynthetic pathways has previously been confirmed in S. aureus but has not been confirmed in other Gram-positive organisms (1, 2). In this study, genes involved in peptidoglycan, teichoic acid, and cell wall exopolysaccharide biosynthesis were upregulated in response to teixobactin (Table S1). Cell wall exopolysaccharides have a role in virulence during enterococcal infections and can increase cell wall density, which may protect against cell wall stress (22). In enterococci, UPP binds activated sugars to form the precursors UPP-MurNAc, UPP-GlcNAc, and UPP-ManNAc of peptidoglycan, teichoic acid, and cell wall exopolysaccharide biosynthesis, respectively (23). We therefore hypothesize that teixobactin targets not only peptidoglycan and teichoic acid biosynthesis but also cell wall exopolysaccharide biosynthesis to effectively inhibit enterococci.

### Comparative analysis of the E. faecalis transcriptional responses to different cell wall-targeting antimicrobials.

Stress response pathways are major contributors to intrinsic antimicrobial resistance (7). Despite this, the cell wall stress response network in enterococci is poorly understood. In order to identify potential pathways that could contribute to intrinsic teixobactin tolerance and, thus, the development of teixobactin resistance, we compared the teixobactin-induced transcriptomic response to previously published E. faecalis transcriptomes of the cell wall-targeting antimicrobials bacitracin, vancomycin, and ampicillin (Table S4) (10). Unsurprisingly, cell wall biogenesis and division, including the biosynthesis of isoprenoid (a precursor of UPP), were the most-upregulated pathways in response to cell wall-targeting antimicrobials (Table S4). Other important components appeared to be two putative autolysins (EF0443 and EF1518), a number of efflux transporters (five ABC transporters and one EmrB-QacA-like MFS transporter), and three TCSs, YclRK (EF1260-EF1261), LiaRS (EF2911-EF2912), and CroRS (EF3289-EF3290) (Table S4). We decided to investigate whether CroRS could have a potential role in teixobactin tolerance.

10.1128/mSphere.00228-19.4 TABLE S4 Comparison of differential gene expression in response to cell wall-targeting antimicrobials teixobactin (Teix), bacitracin (Bac), vancomycin (Van), and ampicillin (Amp) in Enterococcus faecalis. #, 2-fold log_2_ minimum threshold; †, differential gene expression in E. faecalis JH2-2 (this study); ‡, differential gene expression in E. faecalis O1GRF (10). Download Table S4, DOCX file, 0.04 MB. Copyright © 2019 Darnell et al. 2019 Darnell et al. This content is distributed under the terms of the Creative Commons Attribution 4.0 International license.

### The cell wall stress response two-component system CroRS is essential for teixobactin tolerance in E. faecalis.

CroRS is a cell wall stress response TCS, and its role in cephalosporin resistance in enterococci has been well characterized (24, 25). More recently, the CroRS regulon of 219 genes, including an alanine racemase responsible for d-cycloserine resistance, was determined (26). Interestingly, EF0443 (LysM), the second-most-upregulated gene in response to teixobactin, was also characterized as a component of the CroRS regulon (Table 2) (26). To investigate the role of CroRS in teixobactin tolerance, we acquired an E. faecalis JH2-2 *croRS* deletion mutant and compared its MIC and MBC to those of the isogenic wild type (WT), E. faecalis JH2-2 (24). Interestingly, while there was no change in MIC, teixobactin tolerance was completely abolished in the Δ*croRS* mutant, with a decrease in MBC from 16 μg ml^−1^ (WT) to 1 μg ml^−1^ (Δ*croRS* mutant), the same concentration as the observed MIC (Table 3).

**TABLE 3:** Cell antimicrobial MICs and MBCs for the E. faecalis JH2-2 wild type and Δ*croRS* mutant^*a*^

Antimicrobial	WT		Δ*croRS* mutant	
	MIC (μg ml^−1^)	MBC (μg ml^−1^)	MIC (μg ml^−1^)	MBC (μg ml^−1^)
Teixobactin	1	16	1	1
Vancomycin	1	>128	1	1–2
Bacitracin	32	64	16	16
Ampicillin	0.5	2	0.5	2
Gentamicin	32	32	8	8

a Mean MICs and MBCs for at least three biological replicates are reported. Where the MBC values for the biological replicates differed, the MBC ranges are shown. WT, wild type.

**FIG 3:**
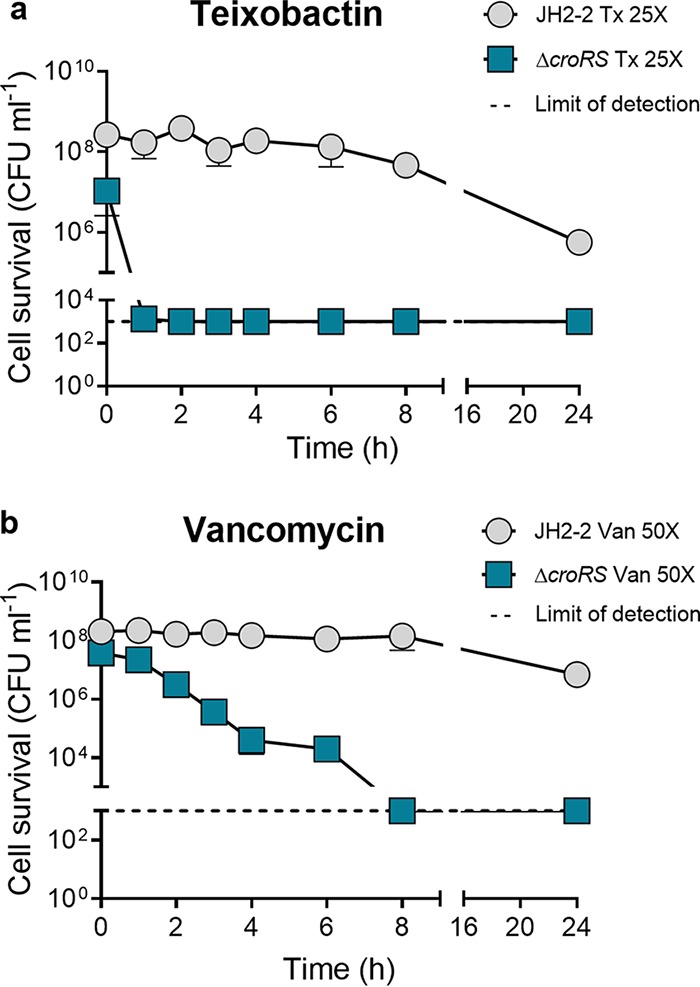
Time-dependent kill kinetic assay of the E. faecalis JH2-2 wild type and Δ*croRS* mutant. Strains were grown to mid-exponential phase (5 × 10^8^ CFU ml^−1^) and untreated or challenged with 25× or 50× MIC of teixobactin (Tx) and vancomycin (Van), respectively. Cell survival (number of CFU ml^−1^) was measured at time zero and 1, 2, 3, 4, 6, 8, and 24 h postchallenge. Results are the mean ± SD (data are for biological triplicates).

To establish whether this was exclusive to teixobactin, we determined the MICs and MBCs for three other cell wall-targeting antimicrobials, vancomycin, bacitracin, and ampicillin, as well as the ribosome-targeting antimicrobial gentamicin, in the Δ*croRS* mutant and compared these to the MICs and MBCs in the WT. We found that tolerance to vancomycin and bacitracin (but not ampicillin) was also completely abolished in the Δ*croRS* mutant, with a decrease in the MBC from >128 μg ml^−1^ (WT) to 1 to 2 μg ml^−1^ (Δ*croRS* mutant) and from 64 μg ml^−1^ (WT) to 16 μg ml^−1^ (Δ*croRS* mutant), respectively (Table 3). Interestingly, the comparative transcriptomic analyses showed that CroRS was upregulated in response to teixobactin, vancomycin, and bacitracin, but not ampicillin (Table S4) (10). No change in the MIC was observed for vancomycin (1 μg ml^−1^) or ampicillin (0.5 μg ml^−1^), while a 2-fold decrease in the MIC (32 to 16 μg ml^−1^) was observed for bacitracin (Table 3). Time-dependent kill kinetics were determined for teixobactin and vancomycin in the Δ*croRS* mutant and compared to those in the WT. These results definitively showed an increase in sensitivity to teixobactin- and vancomycin-induced cell killing in the Δ*croRS* mutant (Fig. 3). The Δ*croRS* mutant was effectively sterilized (5-log reduction) within 2 h of teixobactin challenge (25× MIC), whereas only a 2-log reduction of the WT was seen after 24 h (Fig. 3). For vancomycin, we observed an increase in the sensitivity of the Δ*croRS* mutant with sterilization at 8 h after vancomycin challenge (50× MIC), whereas there was a 1-log reduction of the WT after 24 h (Fig. 3). Surprisingly, a 4-fold reduction in both the MIC and the MBC was observed for gentamicin (Table 3).

Intrinsic low-level resistance to aminoglycosides, like gentamicin, is likely due to an inhibition of the cellular uptake of the antimicrobial (27). The addition of an agent that interferes with cell wall synthesis, such as a β-lactam (ampicillin) or a glycopeptide (vancomycin), greatly increases uptake of the aminoglycoside, enhancing effectivity (27, 28). We hypothesize that the cell wall of a Δ*croRS* mutant is compromised, allowing for an increase in the uptake of gentamicin, resulting in a subsequent decrease in the gentamicin MIC and MBC. This is supported by an increase in sensitivity to glycine, a known destabilizer of the enterococcal cell wall, in the Δ*croRS* mutant compared to the WT (Fig. S3) (29).

10.1128/mSphere.00228-19.9 FIG S3 Glycine assay of the E. faecalis JH2-2 wild type (WT) and Δ*croRS* mutant. SM17 broth (0.5 M sucrose plus M17 medium) containing a range of glycine concentrations was inoculated with an overnight culture of the E. faecalis JH2-2 WT or Δ*croRS* mutant. Cultures were grown overnight at 37°C with no aeration, and growth was measured by determination of the OD_600_. Results are the mean ± SD (data are for biological triplicates). Download FIG S3, TIF file, 0.2 MB. Copyright © 2019 Darnell et al. 2019 Darnell et al. This content is distributed under the terms of the Creative Commons Attribution 4.0 International license.

### Deletion of *lysM* (EF0443) is not sufficient for CroRS-mediated teixobactin tolerance in E. faecalis.

LysM encodes a putative endopeptidase and was the second-most-upregulated gene in response to teixobactin (8.7-fold log_2_ change) (Table S1) (26). Cell wall autolytic enzymes, like endopeptidases, are responsible for regulating normal cell wall turnover; however, their dysregulation can lead to cell lysis (2, 30, 31). In fact, dysregulation of cell wall autolysins via a teixobactin-induced decrease in wall teichoic acids is believed to be the cause of teixobactin-induced cell death in S. aureus (1, 2). As CroRS appears to be important for teixobactin tolerance and is a known regulator of LysM, we sought to determine whether the CroRS-mediated regulation of LysM plays a role in teixobactin tolerance (26). We generated an E. faecalis JH2-2 *ef0443* deletion mutant and subsequently determined the teixobactin MICs and MBCs and compared these to the MICs and MBCs for the WT (32). We observed no difference in the MIC or MBC for the Δ*ef0443* mutant compared to that for the WT (Table S5). This suggests either that LysM does not play an essential role in CroRS-mediated teixobactin tolerance or that more than one factor is required to confer tolerance.

10.1128/mSphere.00228-19.5 TABLE S5 Teixobactin MIC and MBC for E. faecalis JH2-2 Δ*ef0443* compared to those for its isogenic wild type (WT). Mean MICs for three biological replicates are reported. †, MIC; minimum bactericidal concentration. Download Table S5, DOCX file, 0.01 MB. Copyright © 2019 Darnell et al. 2019 Darnell et al. This content is distributed under the terms of the Creative Commons Attribution 4.0 International license.

### Conclusion.

Antimicrobial tolerance is an important preliminary factor in the acquisition of antimicrobial resistance (17). Here we present the teixobactin-induced transcriptome of E. faecalis JH2-2 and isolated CroRS as an important cell wall stress response TCS upregulated by a number of cell wall-targeting antimicrobials. We show that CroRS is an essential regulator of antimicrobial tolerance in E. faecalis; however, the CroRS regulon composes 219 genes, and those responsible for conferring this tolerance are yet to be determined (26).

## MATERIALS AND METHODS

### Bacterial strains and growth conditions.

Enterococcus faecalis strain JH2-2 and variants were routinely grown in brain heart infusion (BHI) broth and agar (1.5%, wt/vol) overnight at 37°C with no aeration unless otherwise stated. Staphylococcus aureus strain ATCC 6538 was routinely grown in tryptic soy broth (TSB) and agar overnight at 37°C with aeration (200 rpm) unless otherwise stated. Cultures for RNA-seq and optimization were grown at a ratio of two-thirds headspace to reduce aeration and minimize bias, as E. faecalis is a facultative anaerobe. Growth was measured as the optical density at a 600-nm wavelength (OD_600_). Teixobactin stocks were made with dimethyl sulfoxide (DMSO) and stored at −20°C.

### Construction of the *ef0443* gene deletion in E. faecalis JH2-2.

An in-frame deletion of *ef0443* was constructed in E. faecalis strain JH2-2 using the pIMAY-Z allelic exchange plasmid, as previously described (32, 33). Primers EF0443_AF, EF0443_BR, EF0443_CF, and EF0443_DR (see Table S6 in the supplemental material) were used to amplify the deletion construct by SLiCE (seamless ligation cloning extract) overlap extension PCR and cloned into the vector by SLiCE. The pIMAY-ZΔ*ef0443* construct was electroporated into JH2-2 using the method of Cruz-Rodz and Gilmore (29).

10.1128/mSphere.00228-19.6 TABLE S6 List of primer sequences used in this study. Download Table S6, DOCX file, 0.01 MB. Copyright © 2019 Darnell et al. 2019 Darnell et al. This content is distributed under the terms of the Creative Commons Attribution 4.0 International license.

### MIC and MBC assays.

Strains were grown to mid-exponential phase (OD_600_, 0.5; 5 × 10^8^ CFU ml^−1^) and were diluted to a final OD_600_ of 0.005 (0.5 McFarland standard). MICs were determined using the broth microdilution method with Muller-Hinton broth in 96-well flat-bottom microtiter plates in accordance with CLSI guidelines. Growth was measured by determination of the OD_600_ using a Varioskan plate reader after 20 h. The MIC was denoted as the lowest inhibitory concentration. Minimum bactericidal concentration (MBCs) were determined by sampling a range of concentrations from a completed MIC plate, followed by serial dilution in 1× phosphate-buffered saline (PBS) and spot plating on BHI or TSB agar (no antibiotic). The plates were incubated at 37°C for 24 h. The MBC was denoted as the lowest concentration that prevented growth on solid agar.

### Time-dependent kill assays.

Time-dependent kill assays were carried out to determine cell death kinetics over time. E. faecalis and S. aureus strains were grown to mid-exponential phase (OD_600_, 0.5; ∼5 × 10^8^ CFU ml^−1^) in BHI and TSB, respectively, and challenged with and without teixobactin (25× MIC or 50× MIC) or vancomycin (50× MIC). Samples were taken at 0, 1, 2, 3, 4, 6, 8, and 24 h postchallenge, serially diluted in 1× PBS, and spot plated on BHI or TSB agar (no antibiotic). The plates were incubated at 37°C for 24 h, and the numbers of CFU ml^−1^ were determined.

### Extraction and preparation of RNA samples for sequence analysis.

To optimize the teixobactin concentration for RNA-seq analysis, E. faecalis JH2-2 cultures were grown in technical duplicate to an OD_600_ of 0.5 and challenged with a range of teixobactin concentrations (0, 0.2, 0.5, and 1 μg ml^−1^) (Fig. S1). DMSO was used as a negative control. The optimal concentration was 0.5 μg ml^−1^.

E. faecalis JH2-2 cultures were grown in technical triplicate to an OD_600_ of 0.5 (37°C, 130 rpm), where each replicate was subsequently split to produce two sets of technical triplicates. One set was challenged with 0.5 μg ml^−1^ of teixobactin, while the other one remained unchallenged. Total RNA was isolated using TRIzol-chloroform extraction as previously described (34). RNA samples were run through an RNeasy minikit (Qiagen) per the manufacturer’s instructions. An Agilent RNA 6000 Nano kit and an Agilent 2100 bioanalyzer (RNA integrity number, >8) were used to verify RNA quality per the manufacturer’s instructions, and the RNA concentration was determined using a NanoDrop ND-100 spectrophotometer.

### RNA sequencing and gene expression analysis.

**(i) cDNA library preparation and sequencing of the Enterococcus faecalis JH2-2 transcriptome.** rRNA was removed from total RNA using a Ribo-Zero RNA removal kit, and cDNA libraries were created using an Illumina TruSeq stranded total RNA library preparation kit. Sequencing was completed using an Illumina MiSeq (v3) system, generating 150-bp single-end reads.

**(ii) Analysis of RNA sequencing data.** Adapter sequences were removed from raw fastq files using Flexbar software (35), and reads shorter than 50 bp were discarded. Sequence reads from each sample were independently mapped to each contig (GenBank accession numbers NZ_KI518257.1 and NZ_KI518256.1) of the E. faecalis JH2-2 genome using Bowtie 2 software (36) to produce a table of raw read counts for all of the JH2-2 genes in each sample. Statistical and principle-component analyses were performed using the Bioconductor DESeq2 package (37). Parameters considered during analysis were the fold change (>2.0-fold log_2_), the mean number of reads (>50), and the adjusted *P* value (*P*_adj_ < 0.1). Genes were also annotated with the E. faecalis V583 (GenBank accession number NC_004668.1) gene homolog using the NCBI BLAST program for continuity. Gene function and ontology were assigned using public databases (NCBI, UniProt, and KEGG) and complemented with literature searches (38–40).

### qRT-PCR of E. faecalis genes in response to teixobactin.

RNA-seq data were validated by quantitative real-time PCR (qRT-PCR) with primers specific to 10 genes, 8 differentially expressed genes and 2 constitutively expressed genes. Gene-specific primers were designed using Primer 3 (v0.4.0) software (http://primer3.ut.ee/ ). Primer sequences can be found in Table S6, and primer concentrations were optimized prior to final validation. cDNA was synthesized using a SuperScript III reverse transcriptase kit (Invitrogen) per the manufacturer’s instructions. cDNA was purified by ethanol precipitation and stored at −20°C. qRT-PCR was carried out using a ViiA 6 real-time PCR system with SYBR green and carboxy-X-rhodamine (Thermo Fisher Scientific). Differential expression was determined using the change in threshold cycle (Δ*C_T_*) values and normalized using the constitutively expressed EF0013 (*dnaB*).

### Glycine assay.

Glycine assays of E. faecalis JH2-2 WT and the Δ*croRS* mutant were carried out in M17 medium with 0.5 M sucrose broth. Cultures were challenged with 0, 3, 5, 6, 7, 8, and 9% glycine and incubated overnight at 37°C with no aeration. Inhibition of growth was measured by determination of the OD_600_ for each strain at each concentration.

### Accession number(s).

The data from this study may be found in ArrayExpress under accession number E-MTAB-6484.
