# Supplementary material for: Genomewide Profiling of the Enterococcus faecalis Transcriptional Response to Teixobactin Reveals CroRS as an Essential Regulator of Antimicrobial Tolerance
Source: mSphere. 2019 May 8;4(3):e00228-19. doi: 10.1128/mSphere.00228-19 (PMC6506618; doi:10.1128/mSphere.00228-19)
Supplement: TABLE S1 [file mSphere.00228-19-st001.docx]

|  | ***E. faecalis*** | |  | **F/C^#^** | |  |  |
| --- | --- | --- | --- | --- | --- | --- | --- |
| **Ontology** | **V583** | **JH2-2** | **Name** | | **Teixobactin** | **Function** | ***p*_adj_** |
|  |  |  |  | |  |  |  |
| **Amino acid metabolism** | | | | | |  |  |
|  | EF0037 | 2864 | *proA* | | 3.0 | γ-glutamyl phosphate reductase | 7.8E-75 |
|  | EF0038 | 2863 | *proB* | | 6.2 | glutamate-5-kinase | 1.62E-29 |
|  | EF0368 | 2596 |  | | 2.7 | aspartate kinase | 4.82E-28 |
|  | EF0634 | 375 |  | | 5.2 | tyrosine decarboxylase | 2.03E-170 |
|  | EF0849 | 581 | *alr* | | 2.2 | alanine racemase | 5.04E-39 |
|  | EF1037 | 768 |  | | 3.8 | aspartate-4-decarboxylase | 9.57E-75 |
|  | EF1133 | 916 | *dapD* | | 4.7 | tetrahydropicolinate succinylase | 5.53E-110 |
|  | EF1134 | 917 |  | | 4.5 | amidohydrolase | 3.83E-112 |
|  | EF1314 | 1109 | *alaT* | | 3.7 | alanine aminotransferase | 4.18E-128 |
|  | EF1561 | 1356 | *aroE* | | 2.1 | shikimate kinase | 1.18E-6 |
|  | EF1731 | 1518 |  | | 2.0 | type I 3-dehydroquinate dehydratase | 1.09E-10 |
|  | EF1793 | 1581 | *ilvE* | | 2.8 | branched chain amino acid transferase | 5.74E-48 |
|  | EF2372 | 1971 | *aspB* | | 2.0 | aspartate aminotransferase | 1.99E-16 |
|  | EF2500 | 2122 |  | | 3.1 | glycine cleavage system protein H | 1.64E-53 |
|  |  |  |  | |  |  |  |
| **Autolysis** | |  |  | |  |  |  |
|  | EF0252 | 2674 |  | | 2.6 | *N*-acetylmuramoyl-L-alanine amidase | 1.01E-38 |
|  | EF0443 | 2523 | *lysM* | | 8.7 | endopeptidase | 1.37E-160 |
|  | EF1518 | 1316 |  | | 9.5 | soluble lytic murein transglycosylase | 2.19E-19 |
|  | EF1583 | 1378 |  | | 2.7 | *N*-acetylmuramoyl-L-alanine amidase | 2.95E-26 |
|  | EF2915 | 2436 | *mltG* | | 3.1 | endolytic murein transglycosylase | 5.11E-94 |
|  |  |  |  | |  |  |  |
| **Cell wall biogenesis and division** | | | | |  |  |  |
|  | EF0668 | 410 | *murE* | | 2.4 | UDP-*N*-acetylmuramoyl-L-alanyl-D-glutamate-L-lysine ligase | 6.09E-32 |
|  | EF0669 | 411 |  | | 3.5 | polysaccharide biosynthesis family protein | 3.06E-84 |
|  | EF0680 | 422 |  | | 5.4 | penicillin binding protein 1A | 3.29E-180 |
|  | EF0746 | 492 |  | | 5.2 | penicillin binding protein | 2.06E-51 |
|  | EF0796 | 540 |  | | 5.2 | type 2 phosphatidic acid phosphatase | 5.65E-35 |
|  | EF0990 | 720 | *ftsL* | | 2.8 | cell division protein | 1.24E-47 |
|  | EF0991 | 721 | *ftsI* | | 2.1 | cell division protein | 3.81E-31 |
|  | EF1027 | 756 |  | | 3.3 | bifunctional lysylphosphatidylglycerol flippase | 1.07E-52 |
|  | EF1111 | 894 |  | | 3.7 | signal peptidase I | 5.35E-58 |
|  | EF1148 | 931 | *pbp1A* | | 3.2 | penicillin binding protein 1AB: peptidoglycan transglycosylase | 1.43E-65 |
|  | EF1169 | 953 | *murAB* | | 2.3 | UDP-*N*-acetylglucosamine 1-carboxyvinyltransferase | 3.56E-14 |
|  | EF1264 | 1048 |  | | 4.0 | phosphotidyl glycerol-membrane oligosaccharide glycerophosphotransferase | 2.74E-133 |
|  | EF1300 | 1084 |  | | 4.9 | putative lipid II flippase | 3.55E-24 |
|  | EF1301 | 1085 |  | | 4.3 | cell cycle protein | 1.57E-23 |
|  | EF1402 | 1190 |  | | 2.4 | putative stimulator FtsZ polymerisation | 1.06E-27 |
|  | EF1546 | 1342 |  | | 2.7 | LysM-containing domain protein | 1.91E-47 |
|  | EF1643 | 1432 | *plsY* | | 2.5 | glycerol-3-phosphate acetyltransferase | 4.57E-13 |
|  | EF1740 | 1528 | *pbp1B* | | 3.3 | penicillin binding protein 1B: peptidase | 4.46E-72 |
|  | EF1813 | 1599 |  | | 3.4 | lipoteichoic acid synthase | 5.93E-30 |
|  | EF1904 | 1670 |  | | 2.9 | glycerophosphoryl diester phosphodiesterase | 1.52E-35 |
|  | EF1908 | 1674 | *murC* | | 2.1 | UDP-*N*-acetylmuramate-L-alanine ligase | 4.18E-32 |
|  | EF1945 | 1706 |  | | 2.9 | putative polyglycerophosphate lipoteichoic acids | 2.2E-15 |
|  | EF2168 | 1874 |  | | 2.1 | lipopolysaccharide choline phosphotransferase | 1.26E-10 |
|  | EF2170 | 1876 | *epaX* | | 3.0 | putative glycosyl transferase family 2 | 1.3E-14 |
|  | EF2174 | 1880 |  | | 2.3 | putative L-alanyl-D-glutamate peptidase | 1.36E-28 |
|  | EF2192 | 1895 | *rfbB* | | 2.3 | dTDP-glucose 4,6-dehydratase | 3.96E-53 |
|  | EF2194 | 1897 | *rfbA* | | 3.0 | glucose-phosphate thymidylyltransferase | 2.42E-104 |
|  | EF2195 | 1898 |  | | 3.5 | α-1,3-L-rhamnosyltransferase | 1.96E-54 |
|  | EF2196 | 1899 | *epaC* | | 4.0 | glycosyl transferase family 2 | 7.1E-66 |
|  | EF2197 | 1900 | *epaB* | | 4.1 | putative α-D-GlcNAc-pyrophosphate polyprenol, α-3-L-rhamnosyl transferase | 7.02E-70 |
|  | EF2198 | 1901 | *tagO* | | 2.5 | undecaprenyl pyrophosphate α-*N*-acetylglucosaminyl 1-phosphate transferase | 1.56E-58 |
|  | EF2439 | 2063 | *uppP* | | 3.8 | undecaprenyl -diphosphatase | 1.4E-37 |
|  | EF2494 | 2114 | *cdsA* | | 2.9 | phosphatidate cytidylyl transferase | 1.8E-61 |
|  | EF2495 | 2115 | *uppS* | | 3.2 | undecaprenyl pyrophosphate synthase | 3.47E-66 |
|  | EF2502 | 2124 |  | | 4.0 | putative cell division protein FtsW | 4.76E-87 |
|  | EF2585 | 2171 | *murT* | | 4.6 | UDP-*N*-acetylmuramyl peptide synthase | 8.71E-161 |
|  | EF2586 | 2172 | *gatD* | | 4.6 | glutamine amidotransferase | 2.94E-137 |
|  | EF2605 | 2190 | *murAA* | | 2.7 | UDP-*N*-acetylglucosamine 1-carboxyvinyltransferase | 1.13E-58 |
|  | EF2658 | 2239 |  | | 4.8 | murM family protein | 2.27E-119 |
|  | EF2691 | 2271 | *plsC* | | 2.7 | 1-acyl-sn-glycerol-3-phosphate acyl transferase | 1.7E-18 |
|  | EF2746 | 2323 | *dltD* | | 2.4 | D-alanyl-lipoteichoic acid biosynthesis | 2.47E-50 |
|  | EF2747 | 2324 | *dltC* | | 2.8 | D-alanine poly(phosphoribitol) ligase subunit 2 | 1.01E-33 |
|  | EF2748 | 2325 | *dltB* | | 3.7 | D-alanyl-lipoteichoic acid biosynthesis | 4.62E-126 |
|  | EF2749 | 2326 | *dltA* | | 4.2 | D-alanine poly(phosphoribitol) ligase | 3.02E-180 |
|  | EF2750 | 2327 | *dltX* | | 5.4 | teichoic acid D-ala incorporation-associated protein | 3.86E-24 |
|  | EF2857 | 2375 | *pbp2B* | | 3.6 | penicillin binding protein 2B | 3.68E-160 |
|  | EF2860 | 2378 |  | | 4.8 | putative peptidoglycan transpeptidase | 2.72E-155 |
|  | EF2882 | 2399 | *fabD* | | 2.2 | malonyl CoA-acyl carrier protein transacylase | 4.37E-29 |
|  | EF2883 | 2400 |  | | 3.4 | enoyl-acyl carrier protein (ACP) reductase | 4.33E-58 |
|  | EF2884 | 2401 | *acpP* | | 2.4 | acyl carrier protein: carries fatty acid chains | 5.69E-43 |
|  | EF2885 | 2402 | *fabH* | | 2.4 | ketoacyl-ACP synthase III | 2.65E-74 |
|  | EF2891 | 2407 | *bgsA* | | 2.8 | glycosyl transferase family 1 | 2.73E-38 |
|  | EF2913 | 1896 | *rfbC* | | 2.7 | dTDP-4-dehydrorhamnose 3,5-epimerase | 3.72E-53 |
|  | EF3060 | 207 | *salA* | | 3.9 | lipase | 1E-97 |
|  | EF3061 | 206 | *mreD* | | 4.8 | cell-shape determining protein | 1.61E-53 |
|  | EF3062 | 205 | *mreC* | | 4.6 | cell-shape determining protein | 9.57E-75 |
|  | EF3245 | 48 |  | | 5.1 | cell-envelope associated acid phosphatase | 2.89E-186 |
|  |  |  |  | |  |  |  |
| **Cofactor biogenesis** | | |  | |  |  |  |
|  | EF0848 | 580 | *acpS* | | 3.3 | holo ACP-synthase | 1.85E-32 |
|  | EF0902 | 632 |  | | 2.5 | phosphomevalonate kinase: IPP biosynthesis | 5.54E-34 |
|  | EF0903 | 633 |  | | 3.2 | diphosphomevalonate decarboxylase: IPP biosynthesis | 4.77E-42 |
|  | EF0904 | 634 |  | | 4.5 | mevalonate kinase: IPP biosynthesis | 1.75E-38 |
|  | EF1225 | 1010 | *apbE* | | 3.1 | lipoprotein: thiamine synthesis | 1.21E-28 |
|  | EF1363 | 1151 |  | | 5.1 | 3-hydroxy-3-methylglutaryl-CoA: IPP biosynthesis | 2.78E-163 |
|  | EF1364 | 1152 |  | | 3.5 | hydroxymethylglutaryl-CoA reductase: IPP biosynthesis | 3.93E-152 |
|  | EF2172 | 1878 |  | | 5.0 | 2-*C*-methyl-D-erythritol 4-phosphate cytidylyltransferase: IPP biosynthesis | 4.17E-50 |
|  |  |  |  | |  |  |  |
| **DNA repair/recombination/replication** | | | | | |  |  |
|  | EF0067 | 2835 | *ruvB* | | 2.2 | Holliday junction DNA helicase | 4.07E-15 |
|  | EF1435 | 932 | *recU* | | 4.3 | Holliday junction-specific endonuclease | 1.07E-61 |
|  | EF1587 | 1382 | *mutT* | | 4.9 | DNA mismatch repair protein | 9.22E-34 |
|  | EF1648 | 1437 | *xerC* | | 4.4 | tyrosine recombinase | 1.21E-119 |
|  | EF2663 | 2242 |  | | 2.3 | ATP-dependent recD-like helicase | 5.33E-25 |
|  |  |  |  | |  |  |  |
| **Metabolism** | |  |  | |  |  |  |
|  | EF0928 | 659 |  | | 3.3 | glucose uptake protein | 1.87E-20 |
|  | EF1411 | 1199 |  | | 3.6 | 6-phospho-β-glucosidase | 3.66E-35 |
|  | EF1618 | 1408 | *eutH* | | 2.0 | ethanolamine utilisation protein | 1.33E-24 |
|  | EF1644 | 1433 | *lacX* | | 2.6 | aldose-1-epimerase | 6.74E-67 |
|  | EF1711 | 1499 |  | | 2.2 | carbonic anhydrase | 8.09E-15 |
|  | EF1741 | 1531 | *ccpA* | | 2.5 | catabolite control protein A | 3.23E-58 |
|  | EF1907 | 1673 |  | | 2.6 | enoyl-CoA hydratase | 1.54E-64 |
|  | EF2171 | 1877 |  | | 3.9 | dTDP-4-dehydro-6-deoxy-D-glucose | 2.05E-10 |
|  | EF2591 | 2177 |  | | 2.9 | glyoxalase | 5.8E-48 |
|  | EF2626 | 2210 |  | | 2.6 | nicotinate phosphoribosyltransferase (NAD scavenging) | 4.79E-38 |
|  |  |  |  | |  |  |  |
| **Protein regulation** | | |  | |  |  |  |
|  | EF1534 | 1330 |  | | 3.7 | peptidyl-prolyl cis-trans isomerase | 1.96E-78 |
|  | EF1646 | 1435 | *hslU* | | 3.3 | heat shock protein HslU-HslV complex | 2.01E-135 |
|  | EF1647 | 1436 | *hslV* | | 3.8 | heat shock protein HslU-HslV complex | 3.47E-85 |
|  | EF2380 | 1978 | *rseP* | | 2.8 | RIP metalloprotease | 3.25E-40 |
|  | EF3282 | 17 | *clpC* | | 2.6 | ATP-dependent Clp protease | 3.7E-70 |
|  |  |  |  | |  |  |  |
| **Purine/pyrimidine metabolism** | | | | |  |  |  |
|  | EF0058 | 2845 | *purR* | | 2.2 | pur operon repressor | 1.55E-12 |
|  | EF0171 | 2750 |  | | 3.6 | adenosine deaminase | 4.73E-25 |
|  | EF0264 | 2662 | *hpt* | | 3.3 | hypoxanthine-guanine phosphoribosyltransferase | 5.32E-54 |
|  | EF0819 | 552 |  | | 3.1 | GTP cyclohydrolase | 1.1E-49 |
|  | EF0825 | 556 |  | | 3.7 | uridine kinase | 3.79E-19 |
|  | EF1547 | 1343 | *cmk* | | 2.4 | cytidylate kinase | 3.26E-47 |
|  | EF2361 | 1960 | *purB* | | 2.4 | adenylsuccinate lyase | 4.14E-44 |
|  | EF2362 | 1961 | *purK* | | 3.4 | 5'-(carboxyamino) imdazole ribonucleotide synthase | 7.06E-56 |
|  | EF2364 | 1963 |  | | 2.3 | xanthine permease | 4.23E-12 |
|  | EF2448 | 2073 |  | | 2.6 | dCMP deaminase: comE operon protein | 1.13E-15 |
|  | EF2555 | 2142 | *tdk* | | 2.0 | thymidine kinase | 1.63E-11 |
|  | EF3127 | 149 |  | | 2.7 | guanylate kinase | 2.74E-32 |
|  | EF3293 | 2943 | *guaB* | | 3.0 | inosine 5'-monophosphate dehydrogenase | 8.36E-42 |
|  |  |  |  | |  |  |  |
| **Resistance/virulence** | | |  | |  |  |  |
|  | EF0737 | 484 |  | | 5.5 | amidase | 2.45E-92 |
|  | EF1371 | 1159 |  | | 2.4 | metallo-β-lactamase | 2.01E-14 |
|  | EF1685 | 1474 | *hlyIII* | | 3.0 | hemolysin III: type II toxin | 1.48E-72 |
|  | EF2216 | 1920 |  | | 3.6 | FUSC family protein | 1.1E-21 |
|  | EF2590 | 2176 |  | | 2.5 | serine hydrolase: β-lactamase transpeptidase family | 1.06E-31 |
|  | EF2698 | 2278 | *telA* | | 3.3 | telA family protein | 1.59E-118 |
|  |  |  |  | |  |  |  |
| **Stress** |  |  |  | |  |  |  |
|  | EF0266 | 2660 |  | | 4.9 | molecular chaperone Hsp33 | 3.44E-39 |
|  | EF0781 | 525 |  | | 2.4 | cold shock protein | 1.08E-18 |
|  | EF1586 | 1381 | *nox* | | 2.5 | NADH oxidase: oxidative stress | 5.87E-104 |
|  | EF1597 | 1391 | *katA* | | 4.5 | catalase/peroxidase katA: oxidative stress | 6.16E-54 |
|  | EF2214 | 1918 |  | | 5.8 | VOC family protein | 1.53E-38 |
|  | EF2501 | 2123 |  | | 3.4 | arsenate reductase: oxidative stress | 2.33E-55 |
|  | EF3164 | 118 | *msrB* | | 2.3 | peptide-methione (R)-S-oxide reductase: oxidative stress | 3.69E-15 |
|  |  |  |  | |  |  |  |
| **Transcriptional regulator** | | | | |  |  |  |
|  | EF0465 | 2501 |  | | 3.4 | LytR family transcriptional regulator | 1.33E-87 |
|  | EF0988 | 718 | *mraZ* | | 2.8 | cell division/cell wall cluster transcriptional repressor | 1.91E-61 |
|  | EF1005 | 735 |  | | 2.5 | iron-dependent transcriptional repressor | 1.4E-21 |
|  | EF1212 | 995 |  | | 3.2 | LytR family transcriptional regulator | 3.78E-79 |
|  | EF1302 | 1086 |  | | 4.2 | transcriptional regulator | 4.08E-38 |
|  | EF1303 | 1087 |  | | 4.6 | LysR family transcriptional regulator | 1.16E-59 |
|  | EF1326 | 1124 |  | | 2.2 | TetR family transcriptional regulator | 2.51E-15 |
|  | EF1525 | 1322 |  | | 2.9 | FUR family transcriptional regulator | 9.6E-61 |
|  | EF1569 | 1364 | *psr* | | 3.7 | putative transcriptional regulator Psr | 1.59E-65 |
|  | EF1599 | 1393 |  | | 5.3 | Cro/cI transcriptional regulator: stress response | 2.16E-16 |
|  | EF1645 | 1434 | *codY* | | 2.9 | GTP-binding transcriptional repressor | 3.93E-95 |
|  | EF1815 | 1601 |  | | 3.5 | LysR family transcriptional regulator | 5.21E-18 |
|  | EF1991 | 1754 | *cspC* | | 2.7 | cold-shock protein transcriptional regulator | 2.31E-20 |
|  | EF2703 | 2285 |  | | 5.3 | LytR family transcriptional regulator | 1.65E-42 |
|  | EF2886 | 2403 |  | | 2.6 | MarR family transcriptional regulator | 4.05E-51 |
|  | EF2913 | 2434 | *liaF* | | 3.7 | cell wall stress response regulator: a component of LiaFSR | 4.52E-64 |
|  | EF3049 | 219 |  | | 3.2 | RpiR family phosphosugar-binding transcriptional regulator | 2.01E-20 |
|  | EF3059 | 208 |  | | 3.2 | TetR family transcriptional regulator | 1.14E-38 |
|  | EF3175 | 105 |  | | 3.8 | putative transcriptional regulator | 2.67E-27 |
|  | EF3283 | 16 | *ctsR* | | 3.2 | CtsR family transcriptional regulator | 4.91E-20 |
|  |  |  |  | |  |  |  |
| **Transcription/translation** | | | | |  |  |  |
|  | EF0262 | 2664 |  | | 2.5 | RNA binding protein | 7.1E-24 |
|  | EF0263 | 2663 |  | | 3.0 | tRNA(ile)-lysine synthase | 1.71E-37 |
|  | EF0267 | 2659 |  | | 3.8 | zinc-binding NifR3 family TIM-barrel protein | 6.86E-47 |
|  | EF0670 | 412 |  | | 4.5 | rRNA pseudouridine synthase | 1.15E-24 |
|  | EF0679 | 421 |  | | 2.1 | RluA family pseudouridine synthase | 1.08E-10 |
|  | EF0701 | 444 | *prfc* | | 2.1 | peptide chain release factor 3 | 1.75E-18 |
|  | EF0794 | 538 |  | | 3.4 | rRNA methyltransferase | 6.09E-52 |
|  | EF0989 | 719 | *rsmH* | | 2.8 | 16s rRNA | 1.11E-59 |
|  | EF1265 | 1049 |  | | 2.7 | RNA binding protein | 4.68E-18 |
|  | EF1527 | 1324 | *obgE* | | 2.5 | GTPase | 1.04E-30 |
|  | EF1649 | 1438 | *gid* | | 2.4 | tRNA(uracil-5-) methyltransferase | 1.66E-24 |
|  | EF2070 | 1836 | *mnmA* | | 2.3 | tRNA-specific-2-thiouridylase | 3.87E-37 |
|  | EF2679 | 2258 | *trpS* | | 2.8 | tryptophanyl-tRNA synthase | 4.39E-34 |
|  | EF2856 | 2374 | *rpmG-3* | | 2.9 | 50S ribosomal protein L33 | 8E-28 |
|  | EF2872 | 2389 |  | | 2.1 | RNA-binding protein | 6.62E-14 |
|  | EF2914 | 2435 | *greA* | | 4.1 | transcription elongation factor | 4.54E-81 |
|  | EF3058 | 209 |  | | 2.5 | phosphotyrosine protein phosphatase | 1.1E-40 |
|  |  |  |  | |  |  |  |
| **Transport/binding** | | |  | |  |  |  |
|  | EF0032 | 2869 |  | | 2.9 | putative copper transporter | 8.76E-63 |
|  | EF0237 | 2688 | *ecfA1* | | 2.1 | energy-coupling factor (ECF) ATPase | 6.35E-19 |
|  | EF0417 | 2550 |  | | 2.7 | ABC transporter permease | 4.67E-24 |
|  | EF0418 | 2549 |  | | 2.5 | ABC transporter ATP-binding protein | 2.53E-17 |
|  | EF0635 | 376 |  | | 4.7 | amino acid permease | 1.55E-105 |
|  | EF0636 | 377 | *nhaC* | | 4.0 | Na+/H+ antiporter | 5.49E-38 |
|  | EF0700 | 443 |  | | 2.1 | HlyC/CorC family transporter | 1.34E-33 |
|  | EF0761 | 507 | *glnH* | | 3.0 | glutamine ABC transporter | 5.26E-29 |
|  | EF0859 | 591 |  | | 3.3 | cation transporter | 1.68E-19 |
|  | EF0872 | 603 |  | | 2.2 | potassium transporter | 1.06E-24 |
|  | EF1135 | 918 | *mscS* | | 2.6 | mechanosensitive ion channel protein | 4.53E-31 |
|  | EF1198 | 982 |  | | 5.7 | ABC transporter permease | 1.03E-49 |
|  | EF1199 | 983 |  | | 4.6 | phosphate ABC transporter: substrate-binding protein | 6.91E-84 |
|  | EF1263 | 1047 |  | | 2.2 | ftsX-like permease | 1.74E-17 |
|  | EF1268 | 1051 |  | | 2.4 | ATPase P: cation transporter E1-E2 family | 1.05E-58 |
|  | EF1304 | 1089 | *mgtA-2* | | 3.7 | magnesium-translocating P-type ATPase | 3.39E-61 |
|  | EF1341 | 1131 | *macA* | | 3.7 | putative macrolide ABC transporter | 1.01E-61 |
|  | EF1492 | 1284 |  | | 2.8 | V-type ATP synthase subunit I | 2.75E-26 |
|  | EF1493 | 1285 |  | | 2.7 | V-type ATP synthase subunit | 8.49E-43 |
|  | EF1519 | 1317 |  | | 3.9 | ATPase: cation transporter E1-E2 family | 2.48E-92 |
|  | EF1541 | 1337 |  | | 2.4 | riboflavin transporter | 1.33E-30 |
|  | EF1672 | 1461 |  | | 4.1 | ABC transporter permease: copper stress | 3.86E-94 |
|  | EF1673 | 1462 |  | | 4.7 | ABC transporter ATP-binding protein: copper stress | 9.42E-25 |
|  | EF1705 | 1492 |  | | 2.5 | phosphate ABC transporter: substrate-binding protein | 3.8E-38 |
|  | EF1727 | 1515 | *ebsA* | | 4.4 | pore-forming protein | 5.59E-19 |
|  | EF1757 | 1546 | *pstA* | | 2.1 | phosphate ABC transporter permease: PTS system | 7.3E-21 |
|  | EF1758 | 1547 | *pstC* | | 2.5 | inorganic phosphate transmembrane transporter | 4.13E-33 |
|  | EF1759 | 1548 |  | | 2.1 | phosphate ABC transporter: substrate-binding protein: PTS system | 3.63E-12 |
|  | EF1760 | 1549 |  | | 2.3 | ABC transporter permease | 4.57E-37 |
|  | EF1761 | 1550 | *ftsE* | | 2.4 | ABC transporter ATP-binding protein: FtsE-like | 2.53E-33 |
|  | EF1763 | 1553 | *secA* | | 2.1 | protein translocase subunit | 2.71E-62 |
|  | EF1814 | 1600 |  | | 4.2 | MFS transporter: drug resistance transporter EmrB-QacA family | 7.48E-31 |
|  | EF2047 | 1813 |  | | 4.2 | amino acid permease: secondary transporter: APC family | 9.13E-21 |
|  | EF2049 | 1816 |  | | 5.1 | peptide ABC transporter: permease | 7.97E-118 |
|  | EF2050 | 1817 |  | | 6.3 | peptide ABC transporter: ATP-binding protein | 7.21E-102 |
|  | EF2183 | 1889 |  | | 3.0 | teichoic acid ABC transporter permease | 1.45E-20 |
|  | EF2460 | 2085 | *typA* | | 2.9 | GTP-binding protein | 9.24E-83 |
|  | EF2498 | 2119 | *metN-2* | | 2.1 | methionine import ATP-binding protein | 6.75E-11 |
|  | EF2601 | 2186 |  | | 2.1 | FMN-dependent NADH-azoreductase: ACP protein | 2.41E-16 |
|  | EF2639 | 2220 |  | | 2.2 | multidrug ABC transporter ATP-binding protein | 6.97E-26 |
|  | EF2721 | 2299 | *sdhb-2* | | 3.2 | L-serine dehydratase, iron-sulfur dependent subunit β | 6.91E-56 |
|  | EF2722 | 2300 | *sdha-2* | | 3.2 | L-serine dehydratase, iron-sulfur dependent subunit α | 3.29E-53 |
|  | EF2873 | 2390 | *yqeH* | | 2.2 | ribosome biogenesis GTPase | 2.82E-28 |
|  | EF2874 | 2391 |  | | 3.0 | haloacid dehalogenase (HAD) superfamily | 5.11E-19 |
|  | EF2910 | 2431 | *trk* | | 2.5 | potassium uptake transporter | 2.54E-46 |
|  | EF2935 | 2456 |  | | 2.8 | xanthine-uracil permease family protein | 4.61E-14 |
|  | EF2985 | 288 |  | | 5.2 | ABC transporter permease | 1.76E-109 |
|  | EF2986 | 287 |  | | 6.8 | ABC transporter ATP-binding protein | 5.18E-51 |
|  | EF2987 | 286 |  | | 6.3 | RND transporter | 7.58E-80 |
|  | EF3041 | 227 |  | | 3.6 | peptide ABC transporter substrate-binding protein | 4.96E-58 |
|  | EF3069 | 199 |  | | 4.2 | formate/nitrite transporter | 3.32E-45 |
|  | no homolog | 1529 |  | | 3.5 | ABC transporter permease | 4.91E-27 |
|  |  |  |  | |  |  |  |
| **Two-component systems** | | | | |  |  |  |
|  | EF1260 | 1044 | *yclR* | | 3.4 | metal-induced stress response regulator | 2.77E-72 |
|  | EF1261 | 1045 | *yclK* | | 2.9 | metal-induced sensor kinase | 2.71E-86 |
|  | EF2911 | 2432 | *liaR* | | 3.6 | luxR family DNA response regulator: a component of LiaFSR | 1.16E-79 |
|  | EF2912 | 2433 | *liaS* | | 3.9 | sensor kinase: a component of LiaFSR | 7.24E-97 |
|  | EF3289 | 2946 | *croR* | | 2.8 | ompR/phoB type DNA-binding response regulator | 1.19E-83 |
|  | EF3290 | 2945 | *croS* | | 2.6 | sensor kinase | 5.19E-106 |
|  |  |  |  | |  |  |  |
| **Unknown function** | | |  | |  |  |  |
|  | EF0026 | 2877 |  | | 4.8 | conserved hypothetical | 2.92E-97 |
|  | EF0261 | 2665 |  | | 2.7 | hypothetical protein | 2.4E-21 |
|  | EF0419 | 2548 |  | | 2.4 | hypothetical protein | 1.9E-29 |
|  | EF0462 | 2504 |  | | 2.1 | hypothetical protein | 4.58E-10 |
|  | EF0468 | 2497 |  | | 3.7 | LemA family protein | 1.38E-22 |
|  | EF0469 | 2496 |  | | 3.6 | hypothetical protein | 1.19E-36 |
|  | EF0708 | 458 |  | | 2.4 | hypothetical protein | 1.13E-29 |
|  | EF0747 | 493 |  | | 3.1 | DUF1003 domain-containing protein | 6.91E-52 |
|  | EF0783 | 528 |  | | 4.8 | acetyltransferase | 3.9E-147 |
|  | EF0795 | 539 |  | | 3.6 | TIGR 01212 family radical SAM protein | 1.76E-77 |
|  | EF0797 | 541 |  | | 5.2 | hypothetical protein | 6.33E-177 |
|  | EF0798 | 542 |  | | 5.4 | hypothetical protein | 3.42E-149 |
|  | EF0802 | 545 |  | | 7.8 | DUF3955 domain-containing protein | 7.48E-71 |
|  | EF0905 | 635 |  | | 2.8 | pentapeptide-repeat containing protein | 1.38E-14 |
|  | EF0931 | 662 |  | | 3.7 | hypothetical protein | 1.79E-18 |
|  | EF0932 | 663 |  | | 7.1 | hypothetical protein | 6.66E-229 |
|  | EF0972 | 703 |  | | 4.0 | metallophosphoesterase | 2.73E-80 |
|  | EF1028 | 757 |  | | 4.9 | α/β hydrolase | 5.67E-47 |
|  | EF1039 | 770 |  | | 4.2 | Cof-type HAD IIB family hydrolase | 3.64E-99 |
|  | EF1063 | 849 |  | | 2.9 | hypothetical protein | 9.05E-19 |
|  | EF1132 | 915 |  | | 4.8 | CBS-domain containing hypothetical protein | 3.46E-87 |
|  | EF1231 | 1016 |  | | 7.6 | metallophosphoesterase | 4.34E-44 |
|  | EF1258 | 1042 |  | | 6.0 | hypothetical protein | 9E-47 |
|  | EF1325 | 1123 |  | | 2.9 | hypothetical protein | 3.31E-23 |
|  | EF1366 | 1154 |  | | 2.1 | hypothetical membrane protein | 5.66E-16 |
|  | EF1403 | 1191 |  | | 2.5 | hypothetical membrane protein | 3.58E-34 |
|  | EF1412 | 1200 |  | | 5.3 | hypothetical protein | 7.78E-91 |
|  | EF1414 | 1202 |  | | 2.2 | hypothetical protein | 3.81E-13 |
|  | EF1532 | 1328 |  | | 6.2 | hypothetical protein | 3.89E-15 |
|  | EF1533 | 1329 |  | | 7.9 | conserved hypothetical protein | 1.31E-65 |
|  | EF1536 | 1332 |  | | 2.9 | α/β hydrolase | 9.98E-18 |
|  | EF1542 | 1338 |  | | 3.3 | hypothetical membrane protein | 4E-34 |
|  | EF1570 | 1365 |  | | 2.9 | DegV family protein | 1.15E-65 |
|  | EF1751 | 1540 |  | | 4.5 | hypothetical protein | 1.79E-190 |
|  | EF1752 | 1541 |  | | 4.3 | hypothetical protein | 1.37E-132 |
|  | EF1753 | 1542 |  | | 4.9 | hypothetical protein | 1.96E-168 |
|  | EF1771 | 1560 |  | | 2.5 | YigZ family protein | 2.79E-18 |
|  | EF1903 | 1669 |  | | 2.8 | hypothetical integral membrane protein | 6E-68 |
|  | EF1905 | 1671 |  | | 2.5 | hypothetical protein | 4.51E-21 |
|  | EF1906 | 1672 |  | | 2.4 | hypothetical integral membrane protein | 6.38E-79 |
|  | EF1909 | 1675 |  | | 2.1 | hypothetical protein | 5.34E-31 |
|  | EF1933 | 1696 |  | | 2.8 | conserved hypothetical protein | 1.54E-19 |
|  | EF1934 | 1697 |  | | 2.6 | hypothetical protein | 1.77E-13 |
|  | EF1946 | 1707 |  | | 3.5 | hypothetical protein | 2.68E-14 |
|  | EF1947 | 1708 |  | | 3.4 | conserved hypothetical protein | 2.31E-34 |
|  | EF2157 | 1863 |  | | 2.3 | DisA-like protein | 3.18E-51 |
|  | EF2169 | 1875 |  | | 2.4 | putative glycosylation ligase | 4.05E-13 |
|  | EF2211 | 1915 |  | | 5.4 | YxeA family protein | 1.09E-40 |
|  | EF2215 | 1919 |  | | 5.2 | hypothetical protein | 3.04E-78 |
|  | EF2373 | 1972 |  | | 2.3 | putative peptidase | 2.03E-22 |
|  | EF2470 | 2091 |  | | 4.3 | putative metal-dependent phosphohydrolase | 9.44E-162 |
|  | EF2499 | 2121 |  | | 3.1 | hypothetical protein | 6.62E-27 |
|  | EF2692 | 2272 |  | | 3.0 | hypothetical protein | 5.72E-20 |
|  | EF2697 | 2277 |  | | 4.1 | hypothetical protein | 2.13E-129 |
|  | EF2740 | 2318 |  | | 2.7 | *O*-methyltransferase | 3.33E-21 |
|  | EF2771 | 2347 |  | | 6.0 | TraX family protein | 5.34E-45 |
|  | EF2784 | 2359 |  | | 2.8 | DUF3042 domain containing protein | 2.13E-51 |
|  | EF2796 | 2371 |  | | 2.2 | hypothetical protein | 3.04E-16 |
|  | EF2862 | 2380 |  | | 4.0 | hypothetical protein | 6.93E-47 |
|  | EF2893 | 2415 |  | | 2.1 | hypothetical protein | 4.41E-20 |
|  | EF2896 | 2418 |  | | 7.2 | DUF3955 domain-containing protein | 1.09E-20 |
|  | EF2909 | 2430 |  | | 2.2 | putative iron-sulfur cluster biosynthesis protein | 3.64E-24 |
|  | EF2929 | 2450 |  | | 4.3 | conserved integral membrane protein | 1.49E-104 |
|  | EF2930 | 2451 |  | | 3.7 | conserved integral membrane protein | 3.98E-34 |
|  | EF3018 | 248 |  | | 3.5 | hypothetical protein | 6.29E-68 |
|  | EF3057 | 210 |  | | 4.0 | hypothetical protein | 3.37E-47 |
|  | EF3078 | 192 |  | | 2.5 | hypothetical protein | 4.91E-16 |
|  | EF3079 | 191 |  | | 3.1 | *N*-acetyltransferase | 7.03E-20 |
|  | EF3149 | 130 |  | | 2.1 | hypothetical protein | 1.38E-45 |
|  | EF3150 | 128 |  | | 3.1 | M16 family peptidase | 1.5E-54 |
|  | EF3151 | 129 |  | | 2.6 | hypothetical protein | 1.22E-40 |
|  | EF3176 | 104 |  | | 4.6 | YccF-domain hypothetical membrane protein | 4.3E-19 |
|  | EF3177 | 103 |  | | 2.8 | hypothetical protein | 1.07E-17 |
|  | EF3192 | 89 |  | | 2.4 | cysteine hydrolase | 5.53E-19 |
|  | no homolog | 1873 |  | | 2.1 | hypothetical protein | 3.61E-11 |
|  | no homolog | 2120 |  | | 3.0 | hypothetical protein | 1.91E-21 |
|  | no homolog | 2876 |  | | 5.0 | hypothetical protein | 2.69E-42 |
|  |  |  |  | |  |  |  |
| **Other** |  |  |  | |  |  |  |
|  | EF0850 | 582 |  | | 2.7 | type II toxin-antitoxin system PemK/MazF toxin | 3.72E-31 |
|  | EF1249 | 1033 | *efba* | | 2.3 | fibronectin/fibrinogen-binding protein | 2.03E-22 |
|  | EF1340 | 1130 |  | | 4.4 | sex pheromone: inducing mate response | 1.08E-40 |
|  | EF1665 | 1454 |  | | 7.6 | conjugal transfer protein TraX | 3.63E-18 |
