# Supplementary material for: Genomewide Profiling of the Enterococcus faecalis Transcriptional Response to Teixobactin Reveals CroRS as an Essential Regulator of Antimicrobial Tolerance
Source: mSphere. 2019 May 8;4(3):e00228-19. doi: 10.1128/mSphere.00228-19 (PMC6506618; doi:10.1128/mSphere.00228-19)
Supplement: TABLE S2 [file mSphere.00228-19-st002.docx]

|  | ***E. faecalis*** | |  | **F/C^#^** |  |  |
| --- | --- | --- | --- | --- | --- | --- |
| **Ontology** | **V583** | **JH2-2** | **Name** | **Teixobactin** | **Function** | ***p*_adj_** |
|  |  |  |  |  |  |  |
| **Amino acid metabolism** | | |  |  |  |  |
|  | EF0104 | 2801 | *arcA* | -7.3 | arginine deaminase | 2.03E-99 |
|  | EF0105 | 2800 | *argF-1* | -6.8 | ornithine carbamoyltransferase | 4.46E-80 |
|  | EF0115 | 2791 |  | -5.2 | imine deaminase | 5.28E-51 |
|  | EF1567 | 1362 | *aroK* | -2.1 | shikimate kinase | 3.34E-19 |
|  | EF1568 | 1363 |  | -2.2 | prephenate dehydratase | 4.24E-33 |
|  | EF2564 | 2150 | *yqeC* | -5.4 | hydroxylase accessory protein | 2.12E-26 |
|  | EF2567 | 2153 | *selD* | -6.2 | selenide water dikinase | 8.67E-31 |
|  | EF2568 | 2154 |  | -7.4 | class V aminotransferase | 1.83E-31 |
|  | EF2581 | 2167 | *ygfK* | -6.5 | putative selenate reductase subunit | 3.24E-63 |
|  | EF3178 | 102 | *dapE* | -2.3 | succinyl-diaminopimelate desuccinylase | 3.33E-26 |
|  |  |  |  |  |  |  |
| **Autolysis** | |  |  |  |  |  |
|  | EF0114 | 2793 |  | -5.5 | glycosyl hydrolase | 3.69E-90 |
|  | EF1348 | 1137 |  | -2.7 | glucan 1,6-α-glucosidase | 3.88E-86 |
|  | EF1349 | 1138 |  | -3.0 | α-glucosidase | 7.72E-120 |
|  | EF1602 | 1395 |  | -6.2 | α-glucosidase | 8.74E-117 |
|  | EF2863 | 2381 |  | -3.1 | endo-β-*N*-acetyl-glucosamine | 1.51E-32 |
|  |  |  |  |  |  |  |
| **Cell envelope biogenesis** | | | |  |  |  |
|  | EF0071 | 2832 |  | -4.3 | putative cell wall-anchored glucosidase | 2.38E-68 |
|  | EF0101 | 317 |  | -3.7 | lysophospholipase | 1.68E-59 |
|  | EF0677 | 419 |  | -4.1 | phosphoglucomutase/phosphomannomutase | 1.44E-56 |
|  | EF1928 | 1692 | *glpO* | -4.9 | α-glycerophosphate oxidase | 7.74E-44 |
|  | EF1929 | 1693 | *glpK* | -5.5 | glycerol kinase | 1.74E-34 |
|  | EF2440 | 2064 |  | -4.9 | celC-like protein: glycan metabolism | 4.18E-42 |
|  | EF2487 | 2107 | *cpsI* | -3.8 | UDP-galactopyranose mutase | 4.86E-42 |
|  | EF2489 | 2109 | *cspG* | -3.1 | UDP-*N*-acetylmuramate dehydrogenase | 2.9E-34 |
|  | EF2644 | 2225 |  | -2.2 | diacylglycerol kinase catalytic subunit | 9.29E-20 |
|  | EF2646 | 2227 |  | -4.6 | glycerate kinase | 3.54E-17 |
|  | EF3005 | 270 |  | -3.3 | choloylglycine hydrolase | 2.72E-47 |
|  |  |  |  |  |  |  |
| **DNA repair/recombination/replication** | | | | |  |  |
|  | EF0004 | 2899 | *recF* | -2.5 | DNA replication and repair protein | 2.96E-68 |
|  | EF0005 | 2898 | *gyrB* | -2.6 | DNA gyrase B subunit | 1.37E-42 |
|  | EF0006 | 2897 | *gyrA* | -2.6 | DNA gyrase A subunit | 1.43E-47 |
|  | EF1874 | 1639 |  | -3.2 | transposase | 2.46E-52 |
|  | EF1874 | 318 |  | -3.8 | IS256 family transposase | 3.26E-29 |
|  |  |  |  |  |  |  |
| **Fatty acid biosynthesis** | | |  |  |  |  |
|  | EF3318 | 2918 | citX | -5.3 | citrate lyase holo-ACP synthase | 8.34E-45 |
|  | EF3319 | 2917 | citF | -5.0 | citrate lyase subunit α | 5.48E-67 |
|  | EF3320 | 2916 | citE | -5.2 | citrate lyase subunit β | 2.21E-92 |
|  | EF3321 | 2915 | citD | -5.2 | citrate lyase ACP | 4.11E-47 |
|  | EF3322 | 2914 | citC | -5.5 | [citrate(pro-3SO-lyase] ligase | 6.46E-88 |
|  |  |  |  |  |  |  |
| **Metabolism** | |  |  |  |  |  |
|  | EF0769 | 514 |  | -2.1 | phosphoenol pyruvate phosphomutase | 3.31E-12 |
| *Carbon* |  |  |  |  |  |  |
|  | EF0174 | 2747 | *deoC* | -2.4 | 2-deoxyribose-5-phosphate 5-dehydrogenase | 2.52E-58 |
|  | EF0185 | 2738 | *deoB* | -2.3 | phosphopentomutase | 2.79E-35 |
|  | EF0253 | 2672 |  | -5.7 | aldehyde dehydrogenase | 1.31E-26 |
|  | EF0271 | 2638 | *arb* | -3.2 | 6-phospho-β-glucosidase | 5.25E-31 |
|  | EF0413 | 2553 |  | -9.7 | mannitol-1-phosphate 5-dehydrogenase | 6.43E-38 |
|  | EF0718 | 467 |  | -2.3 | 1-phosphofructokinase | 1.53E-08 |
|  | EF0960 | 689 |  | -2.0 | maltose 6-phosphate phosphatase | 8.21E-48 |
|  | EF1020 | 749 |  | -5.6 | glycoside hydrolase family I protein | 1.24E-47 |
|  | EF1024 | 754 |  | -2.6 | pyruvate phosphate kinase | 6.16E-28 |
|  | EF1068 | 853 | *galM* | -6.2 | galactose mutarotase: aldose 1-epimerase | 3.61E-75 |
|  | EF1069 | 854 | *galK* | -5.3 | galactokinase | 3.5E-41 |
|  | EF1070 | 855 | *galE-1* | -4.9 | UDP-glucose-4-epimerase | 2.15E-49 |
|  | EF1108 | 891 | *lutA* | -3.9 | oxidoreductase: lactate metabolism | 1.13E-87 |
|  | EF1110 | 893 |  | -3.6 | lactate utilisation protein C | 1.18E-101 |
|  | EF1206 | 990 |  | -6.4 | malate dehydrogenase | 1.62E-58 |
|  | EF1243 | 337 |  | -3.8 | 6-phospho-β-galactosidase | 1.53E-08 |
|  | EF1358 | 1146 | *gldA* | -5.2 | glycerol dehydrogenase | 3.92E-133 |
|  | EF1359 | 1147 | *dhaM* | -4.7 | dihydroxyacetone kinase | 1.46E-100 |
|  | EF1360 | 1148 | *dhaK* | -4.6 | dihydroxyacetone kinase | 8.29E-81 |
|  | EF1361 | 1149 | *dhaL* | -4.6 | oxaloacetate decarboxylase | 6.67E-65 |
|  | EF1511 | 1998 |  | -3.0 | galactonate dehydratase/madalate racemase | 1.11E-19 |
|  | EF1626 | 1419 | *eutL* | -2.2 | carboxysome structural protein | 1.69E-08 |
|  | EF1627 | 1420 | *eutC* | -2.3 | ethanolamine ammonia-lyase light chain | 2.6E-11 |
|  | EF1629 | 1421 | *eutB* | -3.3 | ethanolamine ammonia-lyase heavy chain | 9.37E-23 |
|  | EF1630 | 1422 | *eutA* | -3.9 | ethanolamine utilisation protein | 1.68E-38 |
|  | EF1634 | 1425 | *pduU* | -2.8 | propanedoil utilisation | 2.44E-25 |
|  | EF1635 | 1426 | *pduQ* | -3.4 | alcohol dehydrogenase | 8.19E-39 |
|  | EF1637 | 1427 | *eutT* | -2.8 | cobalamin adenosyltransferase | 7.13E-20 |
|  | EF1638 | 1428 | *eutP/pduV* | -3.1 | ethanolamine utilisation protein | 3.06E-19 |
|  | EF1644 | 338 | *lacX* | -2.3 | aldose 1-epimerase family protein | 3.17E-07 |
|  | EF1707 | 1494 |  | -2.9 | α-mannosidase | 4.24E-45 |
|  | EF1806 | 1592 |  | -2.4 | tagatose-6-phosphate kinase | 4.31E-15 |
|  | EF1806 | 333 |  | -6.5 | tagatose-6-phosphate kinase | 5.56E-78 |
|  | EF1807 | 334 |  | -5.9 | tagatose-bisphosphate aldolase | 7.51E-56 |
|  | EF1834 | 332 | *lacB* | -6.7 | galactose-6-phosphate isomerase subunit | 1.27E-71 |
|  | EF1835 | 331 | *lacA* | -6.4 | galactose-6-phosphate isomerase subunit | 2.92E-64 |
|  | EF1918 | 1683 | *pglA* | -2.3 | 6-phosphoglucanolactonase | 1.6E-42 |
|  | EF1950 | 1711 |  | -4.5 | glucosamine-fructose-6-phosphate aminotransferase | 3.04E-26 |
|  | EF1951 | 1712 |  | -6.0 | glucosamine-fructose-6-phosphate aminotransferase | 2.03E-27 |
|  | EF2151 | 1857 | *glmS* | -2.2 | glutamine-fructose-6 phosphate transaminase | 2.52E-81 |
|  | EF2960 | 2461 | *rbsD* | -4.7 | D-ribose pyranase | 6.09E-51 |
|  | EF2961 | 2462 | *rbsK* | -4.8 | ribokinase | 2.67E-72 |
|  | EF3135 | 142 |  | -6.6 | mannonate dehydratase | 7.99E-15 |
|  | EF3140 | 137 |  | -9.4 | oxidoreductase | 1.48E-40 |
|  | EF3141 | 136 |  | -9.9 | 2-hydroxyacid dehydrogenase | 1.08E-37 |
|  | EF3142 | 135 |  | -9.7 | 6-phosphogluconate dehydrogenase | 1.7E-27 |
|  | EF3157 | 123 |  | -4.5 | glucoside hydrolase family 65 protein | 4.46E-94 |
|  | EF3158 | 122 |  | -4.3 | β-phosphoglucomutase | 9.83E-51 |
|  | EF3316 | 2920 | *maeE* | -2.6 | malate dehydrogenase | 7.36E-15 |
|  | EF3317 | 2919 | *citM* | -4.5 | oxaloacetate decarboxylase | 2.27E-42 |
| *Energy* |  |  |  |  |  |  |
|  | EF2060 | 1826 | *cydB* | -2.7 | cytochrome D ubiquinol oxidase subunit II | 5.24E-53 |
|  | EF2061 | 1827 | *cydA* | -2.9 | cytochrome D ubiquinol oxidase subunit I | 8.31E-62 |
|  | EF2559 | 2145 |  | -6.3 | pyruvate:ferrodoxin oxidoreductase | 6.85E-47 |
|  | EF2560 | 2146 |  | -7.5 | putative glutamate synthase B subunit | 7.34E-23 |
|  | EF2561 | 2147 |  | -6.2 | ferrodoxin-NADP^+^ reductase subunit α | 1.36E-20 |
| *Nitrogen* |  |  |  |  |  |  |
|  | EF0106 | 2799 |  | -6.7 | carbamate kinase I | 1.68E-69 |
|  | EF0386 | 2580 | *arcC-2* | -4.7 | carbamate kinase II | 3.91E-08 |
|  | EF3037 | 229 |  | -2.9 | glutamyl aminopeptidase | 1.02E-40 |
|  |  |  |  |  |  |  |
| **Phage protein** | |  |  |  |  |  |
|  | EF0166 | 2755 |  | -2.5 | phage integrase protein | 9.82E-26 |
|  | EF1284 | 1068 |  | -2.1 | putative phage structural protein | 2.81E-21 |
|  | EF1285 | 1069 |  | -2.2 | phage major tail protein | 8.87E-18 |
|  | EF1290 | 1074 |  | -3.0 | phage structural protein | 1.16E-18 |
|  | EF1291 | 1075 |  | -2.7 | putative prophage protein | 2.54E-23 |
|  |  |  |  |  |  |  |
| **Purine/pyrimidine metabolism** | | | |  |  |  |
|  | EF0062 | 2843 |  | -2.6 | 5' nucleotidase | 7.9E-31 |
|  | EF0173 | 2748 | *deoA* | -2.0 | pyrimidine-nucleoside phosphorylase | 3.52E-49 |
|  | EF0175 | 2746 | *cdd* | -2.4 | cytidine deaminase | 2.75E-37 |
|  | EF0186 | 2737 | *deoD-1* | -2.2 | purine-nucleoside phosphorylase | 1.92E-27 |
|  | EF0187 | 2736 | *deoD-2* | -2.2 | purine-nucleoside phosphorylase | 1.06E-31 |
|  | EF0228 | 2698 | *adk* | -2.8 | adenylate kinase | 7.29E-95 |
|  | EF1921 | 1686 | *rihC* | -6.2 | ribonucleotide hydrolase | 5.09E-29 |
|  | EF2570 | 2156 |  | -6.6 | selenium-dependent xanthine dehydrogenase | 1.13E-76 |
|  | EF2902 | 2423 |  | -2.0 | 2',3'-cyclic-nucleotide 2'-phosphodiesterase | 7.03E-16 |
|  |  |  |  |  |  |  |
| **Stress** |  |  |  |  |  |  |
|  | EF1058 | 844 |  | -2.7 | universal stress protein | 2.34E-17 |
|  | EF1982 | 1744 | *uspA* | -3.4 | universal stress protein | 1.36E-52 |
|  | EF3035 | 231 |  | -2.7 | universal stress protein | 2.58E-24 |
|  | EF3036 | 230 |  | -2.6 | thioredoxin | 7.33E-30 |
|  |  |  |  |  |  |  |
| **Transcriptional regulator** | | |  |  |  |  |
|  | EF0097 | 2808 | *pfoR* | -4.9 | regulatory protein | 0 |
|  | EF0107 | 2798 |  | -5.5 | Crp/Fnr family transcriptional regulator | 4.31E-53 |
|  | EF0663 | 403 |  | -3.2 | YebC/PmpR family DNA-binding transcriptional regulator | 5.7E-22 |
|  | EF0719 | 468 |  | -2.2 | DeoR family transcriptional regulator | 9.53E-11 |
|  | EF0731 | 479 |  | -3.1 | LuxR family transcriptional regulator | 5.75E-21 |
|  | EF1357 | 1145 |  | -3.8 | AraC family transcriptional regulator | 9.27E-40 |
|  | EF1591 | 1385 |  | -5.0 | AraC family transcriptional regulator | 7.11E-40 |
|  | EF1656 | 1445 |  | -5.7 | LysR family transcriptional regulator | 1.14E-27 |
|  | EF1709 | 1496 |  | -3.0 | GntR family transcriptional regulator | 8.2E-57 |
|  | EF1839 | 330 | *lacR* | -2.2 | DeoR/GlpR transcriptional regulator | 1.24E-22 |
|  | EF1955 | 1717 |  | -2.7 | MptR-like transcriptional regulator | 5.13E-25 |
|  | EF2207 | 1911 |  | -2.4 | Fis family transcriptional regulator | 9.08E-20 |
|  | EF2966 | 2467 |  | -9.3 | MltR-like mannitol-operon transcriptional regulator | 8.27E-138 |
|  | EF3193 | 88 | *lrgB* | -5.4 | negative regulator of murein hydrolase activity | 1.43E-113 |
|  | EF3194 | 87 | *lrgA* | -5.0 | negative regulator of murein hydrolase activity | 5.66E-81 |
|  | EF3309 | 2927 | *srlM* | -6.6 | transcriptional anti-terminator | 1.46E-24 |
|  | EF3328 | 2908 | *citO* | -3.6 | GntR family transcriptional regulator | 2.57E-55 |
|  | no homolog | 68 |  | -2.8 | PRD-domain containing transcriptional regulator | 1.39E-28 |
|  |  |  |  |  |  |  |
| **Transcription/translation** | | |  |  |  |  |
|  | EF0003 | 2900 |  | -2.8 | RNA binding protein | 1.15E-40 |
|  | EF0012 | 2892 | *rpII* | -2.1 | 50S ribosomal protein L9 | 1.43E-21 |
|  | EF0100 | 2805 |  | -5.3 | serine-tRNA ligase | 0 |
|  | EF0200 | 2724 |  | -2.0 | elongation factor G | 1.94E-40 |
|  | EF0209 | 2716 | *rplB* | -2.0 | 50S ribosomal protein L2 | 3.33E-33 |
|  | EF0210 | 2715 | *rpsS* | -2.2 | 30S ribosomal protein S19 | 2.3E-33 |
|  | EF0211 | 2714 | *rplV* | -2.2 | 50S ribosomal protein L22 | 1.68E-38 |
|  | EF0212 | 2713 | *rpsQ* | -2.3 | 30S ribosomal protein S3 | 9.1E-76 |
|  | EF0213 | 2712 | *rplP* | -2.3 | 50S ribosomal protein L16 | 5.0E-71 |
|  | EF0214 | 2711 | *rpmC* | -2.3 | 50S ribosomal protein L29 | 3.58E-49 |
|  | EF0215 | 2710 | *rpsQ* | -2.3 | 30S ribosomal protein S3 | 1.46E-46 |
|  | EF0216 | 2709 | *rplN* | -2.3 | 50S ribosomal protein L14 | 4.9E-69 |
|  | EF0217 | 2708 |  | -2.4 | 50S ribosomal protein L24 | 2.82E-67 |
|  | EF0218 | 2707 | *rplE* | -2.4 | 50S ribosomal protein L5 | 4.36E-63 |
|  | EF0220 | 2705 | *rpsH* | -2.5 | 30S ribosomal protein S8 | 9.6E-63 |
|  | EF0221 | 2704 | *rplF* | -2.6 | 50S ribosomal protein L6 | 5.89E-69 |
|  | EF0223 | 2703 | *rplR* | -2.6 | 50S ribosomal protein L18 | 3.48E-78 |
|  | EF0224 | 2702 |  | -2.6 | 30S ribosomal protein S5 | 3.28E-88 |
|  | EF0225 | 2701 | *rpmD* | -2.5 | 50S ribosomal protein L30 | 1.41E-76 |
|  | EF0226 | 2700 | *rplO* | -2.6 | 50S ribosomal protein L15 | 1.68E-85 |
|  | EF1764 | 1555 | *yfiA* | -2.2 | ribosomal subunit interface protein | 8.14E-29 |
|  | EF2205 | 1908 |  | -4.9 | putative 4.5S RNA: signal recognition particle | 8.78E-25 |
|  | EF2715 | 2294 | *rplL* | -2.3 | 50S ribosomal protein L7/L12 | 2.47E-62 |
|  | EF2716 | 2295 | *rplJ* | -2.3 | 50S ribosomal protein L10 | 1.78E-80 |
|  | EF3025 | 240 | *rlmH* | -2.3 | 23s rRNA | 1.11E-22 |
|  | EF3293 | 2944 | *serS-2* | -2.3 | serine-tRNA ligase | 3.34E-51 |
|  |  |  |  |  |  |  |
| **Transport/binding** | |  |  |  |  |  |
|  | EF0098 | 2807 | *sdhB-1* | -5.1 | L-serine dehydratase: iron-sulfur cluster dependent subunit β | 0 |
|  | EF0099 | 2806 | *sdhA-1* | -5.2 | L-serine dehydratase: iron-sulfur cluster dependent subunit α | 0 |
|  | EF0108 | 2797 | *arcD* | -5.0 | C4-dicarboxylate ABC transporter | 1.66E-37 |
|  | EF0176 | 2745 |  | -2.5 | BMP family ABC transporter substrate binding protein: nucleoside import | 2.67E-61 |
|  | EF0177 | 2744 |  | -3.0 | BMP family ABC transporter substrate binding protein: nucleoside import | 4.27E-78 |
|  | EF0178 | 2743 |  | -2.6 | ABC transporter ATP-binding protein: nucleoside import | 1.11E-50 |
|  | EF0179 | 2742 |  | -2.7 | ABC transporter permease: nucleoside import | 7.03E-45 |
|  | EF0180 | 2741 |  | -2.7 | ABC transporter permease | 1.73E-40 |
|  | EF0227 | 2699 | *secY* | -2.6 | preprotein translocase subunit | 8.88E-88 |
|  | EF0385 | 2581 |  | -7.4 | MFS transporter | 6.84E-25 |
|  | EF0387 | 2579 |  | -2.6 | sodium/decarboxylate symporter family | 0.000814 |
|  | EF0706 | 319 |  | -2.8 | ATP-dependent Clp protease ATP-binding | 1.21E-49 |
|  | EF0785 | 530 |  | -2.0 | major facilitator superfamily transporter (ErmB/QacA family protein) | 5.3E-15 |
|  | EF0871 | 601 |  | -2.0 | calcium-translocating P-type ATPase | 7.29E-29 |
|  | EF0875 | 605 |  | -2.6 | copper-translocating P-type ATPase | 5.54E-25 |
|  | EF0938 | 669 |  | -5.5 | sugar ABC transporter ATP-binding protein | 1.44E-169 |
|  | EF1057 | 843 | *mntH2* | -2.9 | manganese transporter | 1.31E-24 |
|  | EF1109 | 892 |  | -3.9 | iron-sulfur cluster binding protein | 1.12E-111 |
|  | EF1207 | 991 | *maeP* | -8.4 | L-malate permease | 6.44E-74 |
|  | EF1639 | 1429 |  | -2.5 | iron ABC transporter: ATP binding protein | 6.23E-36 |
|  | EF1640 | 1430 | *btuC* | -2.6 | iron ABC transporter: permease | 2.01E-51 |
|  | EF1641 | 1431 |  | -2.6 | iron ABC transporter: substrate binding protein | 4.33E-51 |
|  | EF1920 | 1685 |  | -6.8 | C4-dicarboxylate ABC transporter | 2.15E-33 |
|  | EF1927 | 1691 | *glpF* | -5.8 | glycerol uptake facilitator protein | 8.71E-24 |
|  | EF2221 | 1925 |  | -7.4 | ABC transporter substrate-binding protein | 2.32E-42 |
|  | EF2222 | 1926 |  | -8.1 | carbohydrate ABC transporter permease | 2.99E-102 |
|  | EF2223 | 1927 |  | -8.8 | ABC transporter family | 8.39E-91 |
|  | EF2442 | 2066 | *PiT* | -4.9 | inorganic phosphate transporter | 2.77E-24 |
|  | EF2485 | 2105 | *cpsK* | -4.1 | teichoic acid ABC transporter permease | 2.16E-54 |
|  | EF2486 | 2106 | *cpsJ* | -4.1 | sugar ABC transporter ATP-binding protein | 4.48E-107 |
|  | EF2607 | 2191 | *atpC* | -2.1 | ATP synthase epsilon subunit | 4.14E-75 |
|  | EF2647 | 2228 | *grtP* | -5.8 | gluconate-proton symporter | 1.09E-39 |
|  | EF2903 | 2424 |  | -2.1 | sugar ABC transporter substrate binding protein | 1.29E-40 |
|  | EF2959 | 2460 | *rbsU* | -3.9 | ribose uptake protein | 1.95E-76 |
|  | EF2986 | 2003 |  | -4.4 | ABC transporter ATP-binding protein | 6.31E-30 |
|  | EF3106 | 174 |  | -3.6 | oligopeptide ABC transporter | 8.53E-141 |
|  | EF3107 | 173 |  | -2.9 | peptide ABC transporter permease | 5.57E-57 |
|  | EF3108 | 172 |  | -2.3 | ABC transporter permease | 6.48E-35 |
|  | EF3324 | 2912 |  | -5.6 | glutaconyl-CoA carboxylase: biotin carboxyl carrier protein | 1.34E-74 |
|  | EF3325 | 2911 |  | -6.0 | acetyl-CoA carboxylase: biotin carboxyl carrier protein subunit | 1.73E-38 |
|  | EF3327 | 2909 |  | -8.7 | citrate transporter | 5.11E-115 |
| *PTS* |  |  |  |  |  |  |
|  | EF0019 | 2883 | *mptA* | -2.3 | PTS mannose transporter subunit IIB | 4.43E-39 |
|  | EF0020 | 2882 | *mptAB* | -3.2 | PTS mannose transporter subunit IIAB | 1.57E-158 |
|  | EF0021 | 2881 | *mptC* | -3.4 | PTS mannose transporter subunit IIC | 2.75E-180 |
|  | EF0022 | 2880 | *mptD* | -3.5 | PTS mannose transporter subunit IID | 3.15E-210 |
|  | EF0270 | 2639 |  | -4.0 | PTS β-glucoside transporter subunit IIBCA | 5.75E-62 |
|  | EF0411 | 2555 |  | -11.6 | PTS mannitol transporter subunit IICB | 1.05E-99 |
|  | EF0412 | 2554 |  | -10.2 | PTS mannitol transporter subunit IIA | 2.12E-51 |
|  | EF0717 | 466 |  | -3.7 | PTS fructose transporter subunit IIC | 4.26E-23 |
|  | EF0958 | 688 |  | -2.7 | PTS glucose transporter subunit IIABC | 4.12E-88 |
|  | EF1018 | 335 |  | -5.2 | PTS lactose transporter subunit IIA | 6.93E-14 |
|  | EF1019 | 748 |  | -6.7 | PTS sugar transporter subunit IIC | 9.55E-72 |
|  | EF1031 | 742 |  | -8.4 | PTS sugar transporter subunit IIC | 2.11E-28 |
|  | EF1160 | 944 |  | -3.5 | PTS cellobiose transporter subunit IIC | 5.72E-20 |
|  | EF1160 | 336 |  | -4.5 | PTS lactose transporter subunit IIBC | 1.11E-12 |
|  | EF1359 | 962 |  | -4.6 | PTS mannose II A/fructose II A-like transporter subunit | 2.06E-101 |
|  | EF1529 | 1326 |  | -3.4 | PTS sugar transporter subunit IIC | 1.28E-25 |
|  | EF1601 | 1394 |  | -5.7 | PTS β-glucoside transporter subunit IIBCA | 1.65E-76 |
|  | EF1838 | 1999 |  | -3.3 | putative PTS galactitol transporter subunit IIC | 6.5E-24 |
|  | EF1952 | 1713 |  | -7.6 | PTS system mannose/fructose/sorbose family transporter subunit IID | 1.22E-12 |
|  | EF1953 | 1714 |  | -7.7 | PTS sugar transporter subunit IIC | 1.26E-12 |
|  | EF2213 | 1917 |  | -7.3 | PTS maltose transporter subunit IIBC | 4.8E-162 |
|  | EF2964 | 2465 | *ulaA* | -8.9 | PTS ascorbate transporter subunit IIC | 9.61E-23 |
|  | EF2965 | 2466 |  | -9.9 | PTS sugar transporter subunit IIB | 2.13E-20 |
|  | EF3136 | 141 |  | -6.4 | PTS fructose transporter subunit IIA | 8.51E-17 |
|  | EF3137 | 140 |  | -7.8 | PTS mannose/fructose/sorbose transporter subunit IIB | 2.38E-16 |
|  | EF3138 | 139 |  | -9.0 | PTS mannose transporter subunit IID | 2.36E-16 |
|  | EF3139 | 138 |  | -10.9 | PTS sugar transporter subunit IIC | 1.54E-18 |
|  | EF3210 | 72 |  | -8.6 | PTS mannose/fructose/sorbose/*N*-acetylglucosamine subunit IIA | 9.13E-23 |
|  | EF3211 | 71 |  | -9.4 | PTS mannose/fructose/sorbose/*N*-acetylglucosamine subunit IIB | 3.62E-35 |
|  | EF3212 | 70 |  | -8.7 | PTS mannose/fructose/sorbose/*N*-acetylglucosamine subunit IIC | 2.88E-58 |
|  | EF3213 | 69 |  | -9.6 | PTS mannose transporter subunit IID | 2.32E-44 |
|  |  |  |  |  |  |  |
| **Two-component systems** | | |  |  |  |  |
|  | EF1632 | 1423 |  | -2.5 | sensor kinase: ethanolamine utilisation | 1.07E-40 |
|  | EF1633 | 1424 |  | -2.7 | response regulator: ethanolamine utilisation | 6.31E-30 |
|  | EF2219 | 1923 | *yesM* | -2.6 | sensor kinase: transcriptional activator | 1.23E-12 |
|  |  |  |  |  |  |  |
| **Unknown function** | |  |  |  |  |  |
|  | EF0076 | 2829 |  | -2.0 | SDR family oxidoreductase | 7.45E-23 |
|  | EF0095 | 2810 |  | -2.0 | hypothetical protein | 2.15E-21 |
|  | EF0165 | 2756 |  | -2.7 | hypothetical protein | 4.64E-14 |
|  | EF0383 | 2583 | *fdrA* | -7.9 | acyl-CoA synthetase | 2.92E-196 |
|  | EF0384 | 2582 |  | -7.7 | DUF2877 domain-containing protein | 7.31E-59 |
|  | EF0405 | 2561 |  | -4.3 | HAD superfamily hydrolase | 3.22E-82 |
|  | EF0651 | 390 |  | -2.5 | M48 peptidase | 2.18E-20 |
|  | EF0664 | 404 |  | -6.9 | C_GCAxxG_C_C family protein | 1.77E-29 |
|  | EF0678 | 420 |  | -4.1 | *N-*acetyltransferase | 3.69E-36 |
|  | EF0705 | 456 |  | -2.3 | hypothetical protein | 1.51E-24 |
|  | EF0877 | 607 |  | -4.0 | aldo/keto reductase | 9.5E-56 |
|  | EF1021 | 750 |  | -2.2 | GNAT family *N*-acetyltransferase | 4.43E-29 |
|  | EF1075 | 860 |  | -4.6 | *N*-acetyltransferase | 7.56E-27 |
|  | EF1286 | 1070 |  | -2.0 | hypothetical protein | 1.5E-18 |
|  | EF1288 | 1072 |  | -2.8 | hypothetical membrane protein | 2.16E-35 |
|  | EF1350 | 1139 |  | -3.1 | hypothetical membrane protein | 6.05E-29 |
|  | EF1502 | 1294 |  | -2.1 | serine hydrolase | 1.01E-10 |
|  | EF1657 | 1446 |  | -5.4 | hypothetical membrane protein | 4.0E-50 |
|  | EF1708 | 1495 |  | -2.9 | putative glycosyl hydrolase | 4.01E-27 |
|  | EF1919 | 1684 |  | -7.7 | *N*-acetyltransferase | 9.2E-13 |
|  | EF2220 | 1924 |  | -4.5 | hypothetical protein | 1.67E-26 |
|  | EF2566 | 2152 |  | -6.6 | hypothetical protein | 1.3E-19 |
|  | EF2582 | 2168 |  | -9.0 | chlorohydrolase/aminohydrolase | 1.74E-13 |
|  | EF2713 | 2293 |  | -2.1 | LPXTG cell wall anchor domain-containing protein | 1.27E-16 |
|  | EF2785 | 2360 |  | -2.1 | oxidoreductase | 9.36E-18 |
|  | EF2797 | 2372 |  | -2.1 | hypothetical protein | 2.46E-10 |
|  | EF2864 | 2382 |  | -2.7 | putative lipoprotein | 1.58E-19 |
|  | EF3006 | 269 |  | -3.1 | hypothetical protein | 4.56E-29 |
|  | EF3007 | 268 |  | -2.9 | hypothetical protein | 3.06E-37 |
|  | EF3326 | 2910 |  | -6.4 | hypothetical protein | 1.15E-25 |
|  | EF3330 | 2906 |  | -2.1 | protein jag | 8.41E-48 |
|  | no homolog | 305 |  | -2.1 | multicopper oxidase | 4.23E-23 |
|  |  |  |  |  |  |  |
| **Other** |  |  |  |  |  |  |
|  | EF2208 | 1912 | *phzF* | -2.2 | phenazine biosynthesis protein | 4.73E-25 |
|  | EF2569 | 2155 |  | -8.2 | molybdenum cofactor cytidylyltransferase | 2.02E-14 |
|  | no homolog | 267 |  | -4.0 | type I restriction endonuclease subunit R | 1.1E-101 |
