# Supplementary material for: Genomewide Profiling of the Enterococcus faecalis Transcriptional Response to Teixobactin Reveals CroRS as an Essential Regulator of Antimicrobial Tolerance
Source: mSphere. 2019 May 8;4(3):e00228-19. doi: 10.1128/mSphere.00228-19 (PMC6506618; doi:10.1128/mSphere.00228-19)
Supplement: TABLE S3 [file mSphere.00228-19-st003.docx]

|  | Fold-change (log_2_) | |
| --- | --- | --- |
| Gene | qRT-PCR | RNA seq |
| *ef0443* | 8.6 | 8.7 |
| *ef1518* | 8.2 | 9.5 |
| *ef2050* | 6.4 | 6.3 |
| *ef1814* | 3.4 | 4.2 |
| *ef0927* | 0.9 | ND^#^ |
| *ef2198* | 2.5 | 2.5 |
| *ef2911* | 4.0 | 3.6 |
| *ef2912* | 4.6 | 3.9 |
| *ef2913* | 3.6 | 2.7 |
| *ef3120* | -0.2 | ND |
