# Supplementary figures and images for: Genomewide Profiling of the Enterococcus faecalis Transcriptional Response to Teixobactin Reveals CroRS as an Essential Regulator of Antimicrobial Tolerance
Source: mSphere. 2019 May 8;4(3):e00228-19. doi: 10.1128/mSphere.00228-19 (PMC6506618; doi:10.1128/mSphere.00228-19)

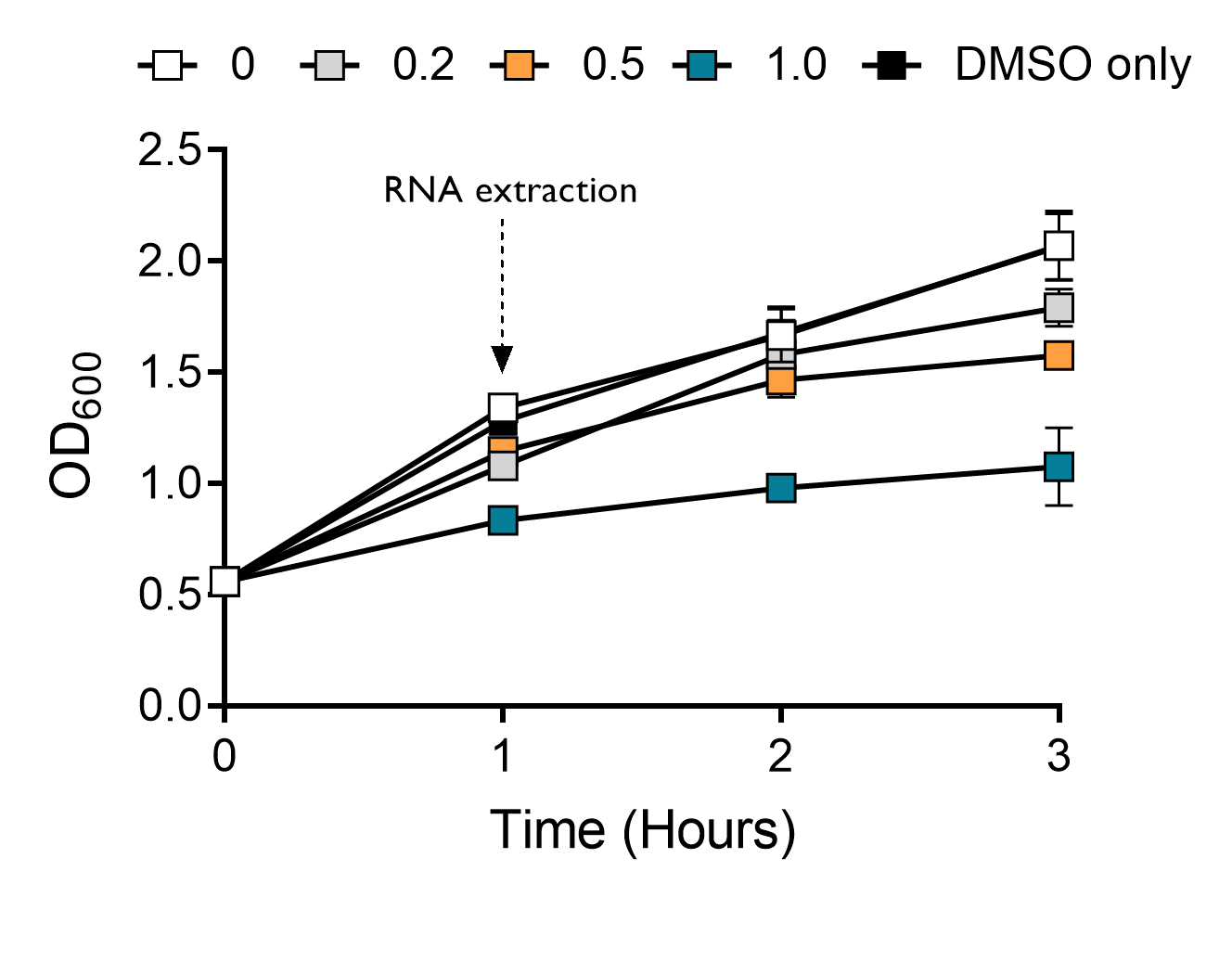

Supplement: FIG S1 [file mSphere.00228-19-sf001.tif]

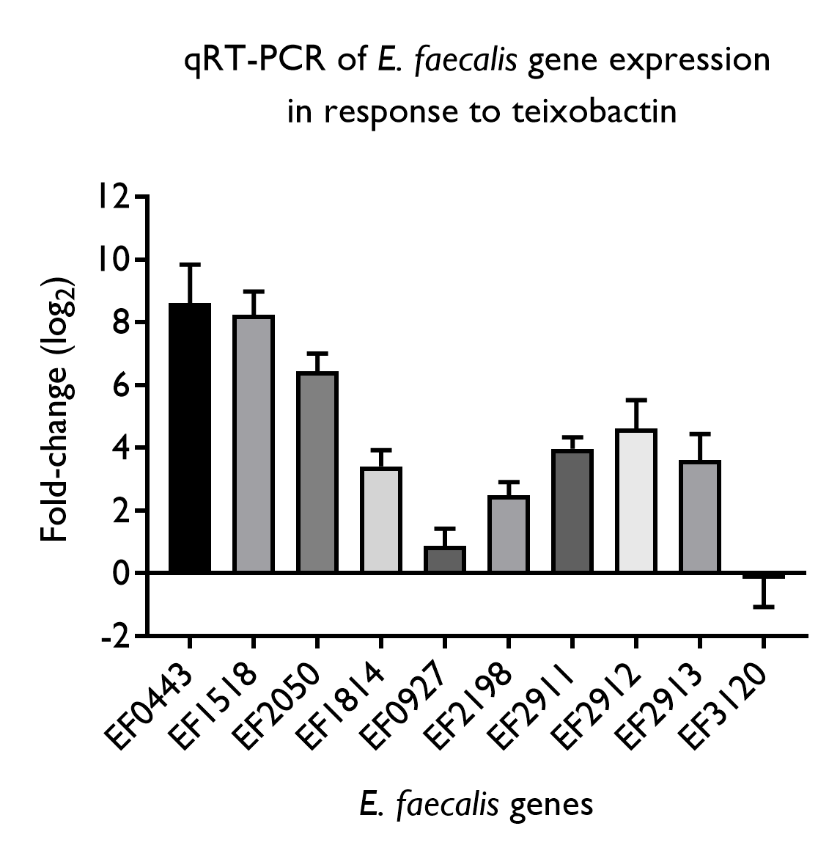

Supplement: FIG S2 [file mSphere.00228-19-sf002.tif]

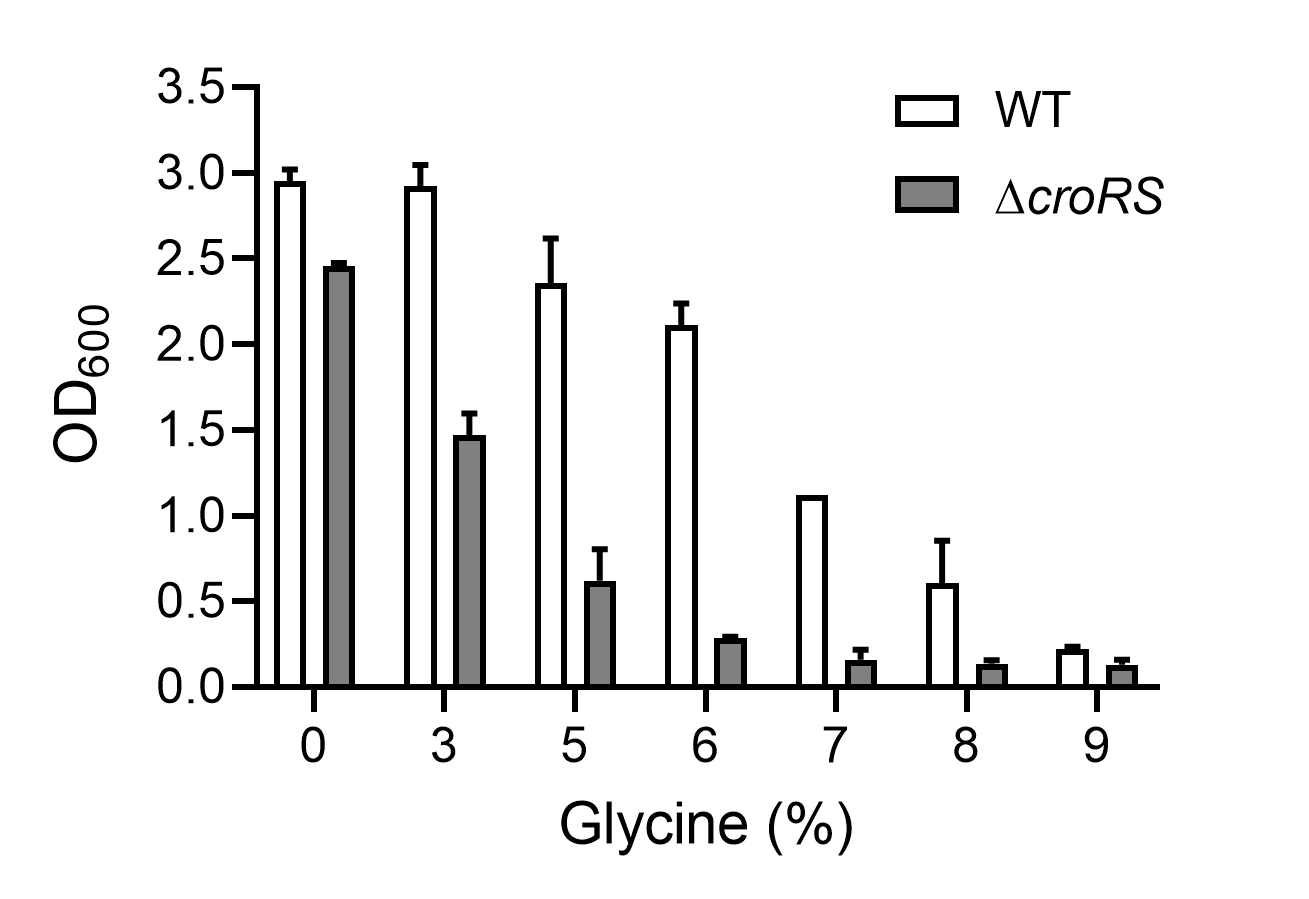

Supplement: FIG S3 [file mSphere.00228-19-sf003.tif]
