# Supplementary material for: Genomewide Profiling of the Enterococcus faecalis Transcriptional Response to Teixobactin Reveals CroRS as an Essential Regulator of Antimicrobial Tolerance
Source: mSphere. 2019 May 8;4(3):e00228-19. doi: 10.1128/mSphere.00228-19 (PMC6506618; doi:10.1128/mSphere.00228-19)
Supplement: TABLE S4 [file mSphere.00228-19-st004.docx]

|  | ***E. faecalis*** | |  | **Fold-change (log_2_)^#^** | | | |  |
| --- | --- | --- | --- | --- | --- | --- | --- | --- |
| **Ontology** | **V583** | **JH2-2** | **Name** | Teix^†^ | **Bac^‡^** | **Van^‡^** | **Amp^‡^** | **Function** |
|  |  |  |  |  |  |  |  |  |
| **Upregulated** | |  |  |  |  |  |  |  |
|  |  |  |  |  |  |  |  |  |
| **Amino acid metabolism** | | | | |  |  |  |  |
|  | EF1314 | 1109 | *alaT* | 3.7 |  | 2.1 |  | alanine aminotransferase |
| **Autolysis** | |  |  |  |  |  |  |  |
|  | EF0443 | 2523 | *lysM* | 8.7 | 4.8 | 6.1 |  | endopeptidase |
|  | EF1518 | 1316 |  | 9.5 |  | 4.3 |  | soluble lytic murein transglycosylase |
| **Cell wall biogenesis and division** | | | |  |  |  |  |  |
|  | EF0680 | 422 |  | 5.4 | 2.8 | 3.7 |  | penicillin binding protein 1A |
|  | EF0746 | 492 |  | 5.2 | 3.1 | 5.6 |  | penicillin binding protein |
|  | EF1264 | 1048 |  | 4.0 | 2.6 | 2.7 |  | phosphotidyl glycerol-membrane oligosaccharide glycerophosphotransferase |
|  | EF1300 | 1084 |  | 4.9 | 3.8 | 5.2 |  | putative lipid II flippase |
|  | EF1301 | 1085 |  | 4.3 | 14.3 |  |  | cell cycle protein |
|  | EF1402 | 1190 |  | 2.4 | 2.0 |  |  | putative stimulator FtsZ polymerisation |
|  | EF1643 | 1432 | *plsY* | 2.5 | -2.1 |  |  | glycerol-3-phosphate acetyltransferase |
|  | EF2192 | 1895 | *rfbB* | 2.3 |  | 2.1 |  | dTDP-glucose 4,6-dehydratase |
|  | EF2194 | 1897 | *rfbA* | 3.0 |  | 2.0 |  | glucose-phosphate thymidylyltransferase |
|  | EF2195 | 1898 |  | 3.5 |  | 2.2 |  | α-1,3-L-rhamnosyltransferase |
|  | EF2196 | 1899 | *epaC* | 4.0 |  | 2.2 |  | glycosyl transferase family 2 |
|  | EF2197 | 1900 | *epaB* | 4.1 |  | 2.3 |  | putative α-D-GlcNAc-pyrophosphate polyprenol, α-3-L-rhamnosyl transferase |
|  | EF2495 | 2115 | *uppS* | 3.2 |  | 2.1 |  | undecaprenyl pyrophosphate synthase |
|  | EF2585 | 2171 | *murT* | 4.6 |  | 2.4 |  | UDP-*N*-acetylmuramyl peptide synthase |
|  | EF2658 | 2239 |  | 4.8 |  | 2.3 |  | murM family protein |
|  | EF2746 | 2323 | *dltD* | 2.4 | 2.2 | 4.2 |  | D-alanyl-lipoteichoic acid biosynthesis |
|  | EF2747 | 2324 | *dltC* | 2.8 | 2.6 | 4.5 |  | D-alanine poly(phosphoribitol) ligase subunit 2 |
|  | EF2749 | 2326 | *dltA* | 4.2 |  | 4.1 |  | D-alanine poly(phosphoribitol) ligase |
|  | EF2750 | 2327 | *dltX* | 5.4 | 2.9 | 4.4 |  | teichoic acid D-ala incorporation-associated protein |
|  | EF2857 | 2375 | *pbp2B* | 3.6 | 2.4 | 3.2 |  | penicillin binding protein 2B |
|  | EF2860 | 2378 |  | 4.8 | 2.1 | 2.2 |  | putative peptidoglycan transpeptidase |
|  | EF2885 | 2402 | *fabH* | 2.4 | 2.3 |  |  | ketoacyl-ACP synthase III |
|  | EF2913 | 1896 | *rfbC* | 2.7 | 2.0 |  |  | dTDP-4-dehydrorhamnose 3,5-epimerase |
|  | EF3060 | 207 | *salA* | 3.9 | 3.4 |  |  | lipase |
|  | EF3245 | 48 |  | 5.1 | 4.9 | 6.8 | 3.1 | cell-envelope associated acid phosphatase |
| **Cofactor biogenesis** | | |  |  |  |  |  |  |
|  | EF0902 | 632 |  | 2.5 |  | 2.7 |  | phosphomevalonate kinase: IPP biosynthesis |
|  | EF0903 | 633 |  | 3.2 |  | 2.8 |  | diphosphomevalonate decarboxylase: IPP biosynthesis |
|  | EF0904 | 634 |  | 4.5 |  | 2.9 |  | mevalonate kinase: IPP biosynthesis |
|  | EF1225 | 1010 | *apbE* | 3.1 |  | 2.8 |  | lipoprotein: thiamine synthesis |
|  | EF1363 | 1151 |  | 5.1 |  | 3.6 |  | 3-hydroxy-3-methylglutaryl-CoA: IPP biosynthesis |
|  | EF1364 | 1152 |  | 3.5 |  | 3.9 |  | hydroxymethylglutaryl-CoA reductase: IPP biosynthesis |
| **DNA repair/recombination/replication** | | | | |  |  |  |  |
|  | EF1587 | 1382 | *mutT* | 4.9 | 3.6 | 4.3 |  | DNA mismatch repair protein |
|  | EF1648 | 1437 | *xerC* | 4.4 |  | 3.5 |  | tyrosine recombinase |
| **Metabolism** | |  |  |  |  |  |  |  |
|  | EF1644 | 1433 | *lacX* | 2.6 |  | 3 |  | aldose-1-epimerase |
|  | EF1907 | 1673 |  | 2.6 |  | 2.2 |  | enoyl-CoA hydratase |
| **Protein regulation** | | |  |  |  |  |  |  |
|  | EF1534 | 1330 |  | 3.7 |  | 2.8 |  | peptidyl-prolyl cis-trans isomerase |
|  | EF1646 | 1435 | *hslU* | 3.3 |  | 3.3 |  | heat shock protein HslU-HslV complex |
|  | EF1647 | 1436 | *hslV* | 3.8 | 2.3 | 3.3 |  | heat shock protein HslU-HslV complex |
| **Purine/pyrimidine metabolism** | | | |  |  |  |  |  |
|  | EF0058 | 2845 | *purR* | 2.2 | -2.3 |  |  | pur operon repressor |
|  | EF0819 | 552 |  | 3.1 |  | 2.5 |  | GTP cyclohydrolase |
|  | EF2364 | 1963 |  | 2.3 |  | -3.5 |  | xanthine permease |
| **Resistance/virulence** | | |  |  |  |  |  |  |
|  | EF2698 | 2278 | *telA* | 3.3 | 3.7 | 3.0 |  | telA family protein |
| **Stress** |  |  |  |  |  |  |  |  |
|  | EF1586 | 1381 | *nox* | 2.5 | 2.2 |  |  | NADH oxidase: oxidative stress |
|  | EF2214 | 1918 |  | 5.8 |  | 3.5 |  | VOC family protein |
| **Transcriptional regulator** | | | |  |  |  |  |  |
|  | EF1212 | 995 |  | 3.2 |  | 2.4 |  | LytR family transcriptional regulator |
|  | EF1302 | 1086 |  | 4.2 | 2.8 | 6.2 |  | transcriptional regulator |
|  | EF1303 | 1087 |  | 4.6 | 3.4 | 7.1 |  | LysR family transcriptional regulator |
|  | EF1569 | 1364 | *psr* | 3.7 |  | 2.8 |  | putative transcriptional regulator Psr |
|  | EF1645 | 1434 | *codY* | 2.9 | 2.3 | 2.9 |  | GTP-binding transcriptional repressor |
|  | EF2703 | 2285 |  | 5.3 | 4.2 | 4.8 |  | LytR family transcriptional regulator |
|  | EF2913 | 2434 | *liaF* | 3.7 | 2.0 |  |  | cell wall stress response regulator: a component of LiaFSR |
|  | EF3059 | 208 |  | 3.2 |  | 3.0 |  | TetR family transcriptional regulator |
| **Transcription/translation** | | | |  |  |  |  |  |
|  | EF1527 | 1324 | *obgE* | 2.5 | -2.1 |  |  | GTPase |
|  | EF2856 | 2374 | *rpmG-3* | 2.9 | 2.2 |  |  | 50S ribosomal protein L33 |
|  | EF3058 | 209 |  | 2.5 |  | 2.7 |  | phosphotyrosine protein phosphatase |
| **Transport/binding** | | |  |  |  |  |  |  |
|  | EF0032 | 2869 |  | 2.9 |  | 2.5 |  | putative copper transporter |
|  | EF1135 | 918 | *mscS* | 2.6 |  | 2.0 |  | mechanosensitive ion channel protein |
|  | EF1199 | 983 |  | 4.6 |  | 3.8 |  | phosphate ABC transporter: substrate-binding protein |
|  | EF1263 | 1047 |  | 2.2 |  | 4.4 |  | ftsX-like permease |
|  | EF1268 | 1051 |  | 2.4 |  | 3.8 |  | ATPase P: cation transporter E1-E2 family |
|  | EF1304 | 1089 | *mgtA-2* | 3.7 | 4.0 | 6.3 |  | magnesium-translocating P-type ATPase |
|  | EF1672 | 1461 |  | 4.1 |  | 2.7 |  | ABC transporter permease: copper stress |
|  | EF1673 | 1462 |  | 4.7 | 2.5 |  |  | ABC transporter ATP-binding protein: copper stress |
|  | EF1814 | 1600 |  | 4.2 | 3.0 |  | 2.6 | MFS transporter: drug resistance transporter EmrB-QacA family |
|  | EF2050 | 1817 |  | 6.3 | 2.4 |  |  | peptide ABC transporter: ATP-binding protein |
|  | EF2183 | 1889 |  | 3.0 |  | 2.3 |  | teichoic acid ABC transporter permease |
|  | EF2722 | 2300 | *sdha-2* | 3.2 |  | 2.7 |  | L-serine dehydratase, iron-sulfur dependent subunit α |
|  | EF2935 | 2456 |  | 2.8 | -2.2 |  |  | xanthine-uracil permease family protein |
|  | EF2985 | 288 |  | 5.2 | 2.8 |  |  | ABC transporter permease |
|  | EF2986 | 287 |  | 6.8 |  | 2.6 |  | ABC transporter ATP-binding protein |
|  | EF2987 | 286 |  | 6.3 | 2.0 |  |  | RND transporter |
| **Two-component systems** | | | |  |  |  |  |  |
|  | EF1260 | 1044 | *yclR* | 3.4 |  | 2.4 |  | metal-induced stress response regulator |
|  | EF1261 | 1045 | *yclK* | 2.9 |  | 2.2 |  | metal-induced sensor kinase |
|  | EF2911 | 2432 | *liaR* | 3.6 | 2.0 |  |  | luxR family DNA response regulator: a component of LiaFSR |
|  | EF2912 | 2433 | *liaS* | 3.9 | 2.8 |  |  | sensor kinase: a component of LiaFSR |
|  | EF3289 | 2946 | *croR* | 2.8 | 2.6 | 3.7 |  | ompR/phoB type DNA-binding response regulator |
|  | EF3290 | 2945 | *croS* | 2.6 |  | 3.7 |  | sensor kinase |
| **Unknown function** | | |  |  |  |  |  |  |
|  | EF0026 | 2877 |  | 4.8 | 2.9 | 3.6 | 3.2 | conserved hypothetical |
|  | EF0462 | 2504 |  | 2.1 | -2.7 |  |  | hypothetical protein |
|  | EF0468 | 2497 |  | 3.7 | 2.3 |  |  | LemA family protein |
|  | EF0469 | 2496 |  | 3.6 | 2.2 |  |  | hypothetical protein |
|  | EF0708 | 458 |  | 2.4 | 2.6 | 4.0 |  | hypothetical protein |
|  | EF0747 | 493 |  | 3.1 | 2.9 |  |  | DUF1003 domain-containing protein |
|  | EF0783 | 528 |  | 4.8 |  | 4.0 |  | acetyltransferase |
|  | EF0797 | 541 |  | 5.2 | 3.7 | 3.4 | 3.0 | hypothetical protein |
|  | EF0798 | 542 |  | 5.4 | 4.0 | 3.2 |  | hypothetical protein |
|  | EF0802 | 545 |  | 7.8 | 4.0 | 7.9 | 2.6 | DUF3955 domain-containing protein |
|  | EF0932 | 663 |  | 7.1 |  | 4.2 |  | hypothetical protein |
|  | EF0972 | 703 |  | 4.0 | 3.4 | 6.2 |  | metallophosphoesterase |
|  | EF1132 | 915 |  | 4.8 |  | 2.9 |  | CBS-domain containing hypothetical protein |
|  | EF1231 | 1016 |  | 7.6 |  | 6.1 |  | metallophosphoesterase |
|  | EF1258 | 1042 |  | 6.0 | 3.5 | 4.4 |  | hypothetical protein |
|  | EF1533 | 1329 |  | 7.9 | 5.2 | 4.4 | 3.5 | conserved hypothetical protein |
|  | EF1751 | 1540 |  | 4.5 | 2.0 | 3.1 |  | hypothetical protein |
|  | EF1752 | 1541 |  | 4.3 | 2.1 | 3.4 |  | hypothetical protein |
|  | EF1753 | 1542 |  | 4.9 | 4.2 | 4.2 | 2.0 | hypothetical protein |
|  | EF1906 | 1672 |  | 2.4 |  | 2.6 |  | hypothetical integral membrane protein |
|  | EF1933 | 1696 |  | 2.8 | 2.8 |  |  | conserved hypothetical protein |
|  | EF1946 | 1707 |  | 3.5 | 2.2 |  |  | hypothetical protein |
|  | EF1947 | 1708 |  | 3.4 | 2.6 |  |  | conserved hypothetical protein |
|  | EF2215 | 1919 |  | 5.2 |  | 3.1 |  | hypothetical protein |
|  | EF2470 | 2091 |  | 4.3 |  | 2.7 |  | putative metal-dependent phosphohydrolase |
|  | EF2697 | 2277 |  | 4.1 | 2.8 | 2.9 |  | hypothetical protein |
|  | EF2784 | 2359 |  | 2.8 | 3.6 |  |  | DUF3042 domain containing protein |
|  | EF2896 | 2418 |  | 7.2 | 4.9 | 6.6 | 2.6 | DUF3955 domain-containing protein |
|  | EF3018 | 248 |  | 3.5 | 2.0 |  |  | hypothetical protein |
|  | EF3057 | 210 |  | 4.0 |  | 2.9 | 2.7 | hypothetical protein |
| **Other** |  |  |  |  |  |  |  |  |
|  | EF1340 | 1130 |  | 4.4 | 2.8 |  |  | sex pheromone: inducing mate response |
|  |  |  |  |  |  |  |  |  |
| **Downregulated** | |  |  |  |  |  |  |  |
|  |  |  |  |  |  |  |  |  |
| **Cell envelope biogenesis** | | | |  |  |  |  |  |
|  | EF0101 | 317 |  | -3.7 | -2.5 |  |  | lysophospholipase |
|  | EF2440 | 2064 |  | -4.9 |  | -2.8 |  | celC-like protein: glycan metabolism |
| **Metabolism** | |  |  |  |  |  |  |  |
| *Carbon* |  |  |  |  |  |  |  |  |
|  | EF1511 | 1998 |  | -3.0 |  | 2.9 |  | galactonate dehydratase/madalate racemase |
|  | EF1644 | 338 | *lacX* | -2.3 |  | 3.0 |  | aldose 1-epimerase family protein |
|  | EF1707 | 1494 |  | -2.9 |  | 2.4 |  | α-mannosidase |
| **Transcriptional regulator** | | |  |  |  |  |  |  |
|  | EF0107 | 2798 |  | -5.5 | -2.2 |  |  | Crp/Fnr family transcriptional regulator |
|  | EF1656 | 1445 |  | -5.7 |  | 2.8 |  | LysR family transcriptional regulator |
|  | EF3193 | 88 | *lrgB* | -5.4 | 3.0 |  |  | negative regulator of murein hydrolase activity |
| **Transport/binding** | |  |  |  |  |  |  |  |
|  | EF0108 | 2797 | *arcD* | -5.0 | -2.3 |  |  | C4-dicarboxylate ABC transporter |
|  | EF0177 | 2744 |  | -3.0 |  | -2.3 |  | BMP family ABC transporter substrate binding protein: nucleoside import |
|  | EF2647 | 2228 | *grtP* | -5.8 | -2.5 |  |  | gluconate-proton symporter |
|  | EF3327 | 2909 |  | -8.7 | -3.3 |  |  | citrate transporter |
| *PTS* |  |  |  |  |  |  |  |  |
|  | EF0958 | 688 |  | -2.7 | -2.9 |  |  | PTS glucose transporter subunit IIABC |
|  | EF3136 | 141 |  | -6.4 |  | 2.4 |  | PTS fructose transporter subunit IIA |
|  | EF3210 | 72 |  | -8.6 |  | -2.9 |  | PTS mannose/fructose/sorbose/*N*-acetylglucosamine subunit IIA |
|  | EF3211 | 71 |  | -9.4 |  | -2.6 |  | PTS mannose/fructose/sorbose/*N*-acetylglucosamine subunit IIB |
| **Unknown function** | |  |  |  |  |  |  |  |
|  | EF0095 | 2810 |  | -2.0 | -4.1 |  |  | hypothetical protein |
|  | EF0664 | 404 |  | -6.9 | -2.1 |  |  | C_GCAxxG_C_C family protein |
|  | EF1657 | 1446 |  | -5.4 | -2.6 |  |  | hypothetical membrane protein |
| **Other** |  |  |  |  |  |  |  |  |
|  | EF2569 | 2155 |  | -8.2 | -2.1 |  |  | molybdenum cofactor cytidylyltransferase |
