# Supplementary material for: Genomewide Profiling of the Enterococcus faecalis Transcriptional Response to Teixobactin Reveals CroRS as an Essential Regulator of Antimicrobial Tolerance
Source: mSphere. 2019 May 8;4(3):e00228-19. doi: 10.1128/mSphere.00228-19 (PMC6506618; doi:10.1128/mSphere.00228-19)
Supplement: TABLE S5 [file mSphere.00228-19-st005.docx]

|  | **MIC^†^ (μg ml^-1^)** | **MBC^†^ (μg ml^-1^)** |
| --- | --- | --- |
| WT | 2 | 16 |
| Δ*ef0443* | 2 | 16 |
