# Supplementary material for: Genomewide Profiling of the Enterococcus faecalis Transcriptional Response to Teixobactin Reveals CroRS as an Essential Regulator of Antimicrobial Tolerance
Source: mSphere. 2019 May 8;4(3):e00228-19. doi: 10.1128/mSphere.00228-19 (PMC6506618; doi:10.1128/mSphere.00228-19)
Supplement: TABLE S6 [file mSphere.00228-19-st006.docx]

| **Primer name** | **Sequence** | **Purpose** |
| --- | --- | --- |
| EF0013_Left | GTAGTTTGTCCCGTGCGAAT | qRT-PCR |
| EF0013_Right | CTGTTGGCGGTTCTCTTTTC | qRT-PCR |
| EF0443_Left | GCAGTGATGCAAAAGAGTGG | qRT-PCR |
| EF0443_Right | ATGAACCATAGCGACCTGCT | qRT-PCR |
| EF0927_Left | TTTTAACCGACCGAAAATGG | qRT-PCR |
| EF0927_Right | AATGCGTCGTTGATCTTGTG | qRT-PCR |
| EF1518_Left | GCTGGTCTTGGCTATCTTGG | qRT-PCR |
| EF1518_Right | GACCCCATGTGCGATACTTT | qRT-PCR |
| EF1814_Left | GGCGAAATGGTAGGGAGAAT | qRT-PCR |
| EF1814_Right | GCCGTTCCTGACTGAGAAAG | qRT-PCR |
| EF2050_Left | AAGGATGTGATGGGCTTGTT | qRT-PCR |
| EF2050_Right | TGGTTGCCGTTTTGGATAAT | qRT-PCR |
| EF2198_Left | TTCCTCGGGTTTATGATTGC | qRT-PCR |
| EF2198_Right | GGAAATGGGCTTCTTGTTCA | qRT-PCR |
| EF2911_Left | TCGTAGGCGAAGCAGAAAAT | qRT-PCR |
| EF2911_Right | CAATCGCCGGATACACTTT | qRT-PCR |
| EF2912_Left | AATGGATGGGCAAACAAAAG | qRT-PCR |
| EF2912_Right | CTGATAGGACGCAAGTGCAA | qRT-PCR |
| EF2913_Left | TGCTCGCTCAGCTATATCCA | qRT-PCR |
| EF2913_Right | GGCGTTTTACTTGTGGCCTA | qRT-PCR |
| EF3120_Left | AGCATGGCGACTAACCAATC | qRT-PCR |
| EF3120_Right | TGACGAACCACATTTTCCAA | qRT-PCR |
| EF0443_AF | CCTCACTAAAGGGAACAAAAGCTGGGTACCAGTTGCTTTGGGGAGACCTAAACC | EF0443 knockout |
| EF0443_BR | CATGTATGTTTCTCCTTTGTAATTAAAAATATTTG | EF0443 knockout |
| EF0443_CF | ATATTTTTAATTACAAAGGAGAAACATACATGTAAAATTAAATAAACGAAGTAATCTAATTACT | EF0443 knockout |
| EF0443_DR | CGACTCACTATAGGGCGAATTGGAGCTCAAATCACAAACTAATTGTAAAACATTGC | EF0443 knockout |
